# Biochemical phenotyping of multiple myeloma patients at diagnosis reveals a disorder of mitochondrial complexes I and II and a Hartnup-like disturbance as underlying conditions, also influencing different stages of the disease

**DOI:** 10.1038/s41598-020-75862-4

**Published:** 2020-12-14

**Authors:** Ismael Dale Cotrim Guerreiro da Silva, Erica Valadares de Castro Levatti, Amanda Paula Pedroso, Dirce Maria Lobo Marchioni, Antonio Augusto Ferreira Carioca, Gisele Wally Braga Colleoni

**Affiliations:** 1 Departament of Gynecology, Paulista School of Medicine, Federal University of São Paulo, São Paulo, Brazil; 2 Department of Clinical and Experimental Oncology, Paulista School of Medicine, Federal University of São Paulo, São Paulo, Brazil; 3 Departament of Physiology, Paulista School of Medicine, Federal University of São Paulo, São Paulo, Brazil; 4 Nutrition Department, School of Public Health, University of São Paulo (MUSP), São Paulo, Brazil; 5 Nutrition Department, University of Fortaleza (UNIFOR), Fortaleza, Brazil

**Keywords:** Biochemistry, Cancer

## Abstract

The aim of this study was to identify novel plasma metabolic signatures with possible relevance during multiple myeloma (MM) development and progression. A biochemical quantitative phenotyping platform based on targeted electrospray ionization tandem mass spectrometry technology was used to aid in the identification of any eventual perturbed biochemical pathway in peripheral blood plasma from 36 MM patients and 73 healthy controls. Our results showed that MM cases present an increase in short and medium/long-chain species of acylcarnitines resembling Multiple AcylCoA Dehydrogenase Deficiency (MADD), particularly, associated with MM advanced International Staging System (ISS). Lipids profile showed lower concentrations of phosphatidylcholine (PC), lysophosphatidylcholine (LPC) and sphingomyelins (SM) in the MM patients and its respective ISS groups. MM cases were accompanied by a drop in the concentration of essential amino acids, especially tryptophan, with a significant inverse correlation between the progressive drop in tryptophan with the elevation of β2-microglobulin, with the increase in systemic methylation levels (Symmetric Arginine Dimethylation, SDMA) and with the accumulation of esterified carnitines in relation to free carnitine (AcylC/C0). Serotonin was significantly elevated in cases of MM, without a clear association with ISS. Kynurenine/tryptophan ratio demonstrates that the activity of dioxigenases is even higher in the cases classified as ISS 3. In conclusion, our study showed that MM patients at diagnosis showed metabolic disorders resembling both mitochondrial complexes I and II and Hartnup-like disturbances as underlying conditions, also influencing different stages of the disease.

## Introduction

Multiple myeloma (MM) is characterized by proliferation and infiltration of clonal plasma cells into the bone marrow microenvironment, which produce and secrete monoclonal immunoglobulin that can be detected in patients' urine or serum^1^ (Palumbo and Anderson, 2011). Symptoms associated with MM correspond to hypercalcemia (C), renal failure (R), anemia (A) and bone lesions (B), manifestations known as CRAB symptoms^2^. MM is the second most common onco-hematological disease in the world, and an increase in its incidence has been observed due to the increase in life expectancy of the world population^2^.

The cause of MM is still unknown. There are a few risk factors that could increase the chance of MM development such as: (1) increasing age (less than 1% of MM cases are diagnosed in patients under 35 years old and most of them occur after 65 years); (2) MM is more frequently diagnosed in men than women; (3) it is also more common in African than in white Americans; (4) MM can be diagnosed in more than one member in some families. (5) apparently, obesity increases the risk of developing MM^3,4^. Case-controlled studies have suggested a significant risk of developing MM in individuals with important occupational exposures in the agriculture, food, and petrochemical industries, especially in patients exposed to herbicides or insecticides and to benzene and other organic solvents^4,5^.

MM is known to begin with a premalignant phase known as monoclonal gammopathy of undetermined significance (MGUS), but the biological mechanisms for disease progression are not yet fully understood^6^. MGUS is found in less than 5% of individuals under 50 years old and in high percentage in older populations. Disease progression can be defined based on some clinical criteria and risk stratification models have been developed^6,7^: one of them is known as the International Staging System (ISS), as a function of serum β2-microglobulin and albumin values (ISS I, < 3.5 mg/L β2-microglobulin and ≥ 3.5 g/dL albumin; ISS II, < 3.5 mg/L β2-microglobulin and < 3.5 g/dL albumin or 3.5 to < 5.5 mg/L β2-microglobulin; ISS III, > 5.5 mg/L β2-microglobulin)^8^.

Despite advances in the treatment of MM due to the development of new drugs^9,10^ most patients are in the intermediate or late stages when the disease is diagnosed. Thus, development of new drugs is needed, as the incidence of MM increases annually^11^.

Proteomics and metabolomics analysis have contributed significantly with new insights in cancer biology, since both approaches contribute to a better understanding of the disease, diagnosis, classification, treatment decision, treatment efficacy evaluation and identification of new therapeutic targets^10–14^.

MM is an incurable disease and there are still few studies on its metabolome^11,13–15^. It has already been possible to observe that patients diagnosed with MM have high plasma or serum levels of some amino acids such as isoleucine, arginine, phenylalanine and tyrosine, as well as some lipids, when compared to normal controls^14^. Decreased tryptophan plasma or serum levels were observed in patients with MM and appear to be associated with activation of the kynurenine pathway by increasing indolamine 2,3-dioxigenase 1 (IDO-1) activity. Decreased levels of some lysophosphatidylcholines and carnitines were also detected^14^.

Since cancer progression can involve metabolism reprogramming^16^ metabolomics seems to be an excellent tool for better understanding the molecular basis of MM and new pathways involved in disease progression.

Therefore, the aim of this study was to identify novel plasma biomarkers and metabolic signatures with possible relevance during MM progression. A biochemical quantitative phenotyping platform based on targeted electrospray ionization tandem mass spectrometry technology (ESI–MS/MS) (Biocrates, p180) was used to aid in the identification of any eventual perturbed biochemical pathway in peripheral blood plasma from MM patients and healthy controls.

## Results

### Patients with MM exhibit biochemical changes associated with a worse prognosis, which suggest disorder in mitochondrial complexes I and II

**Figure 1:**
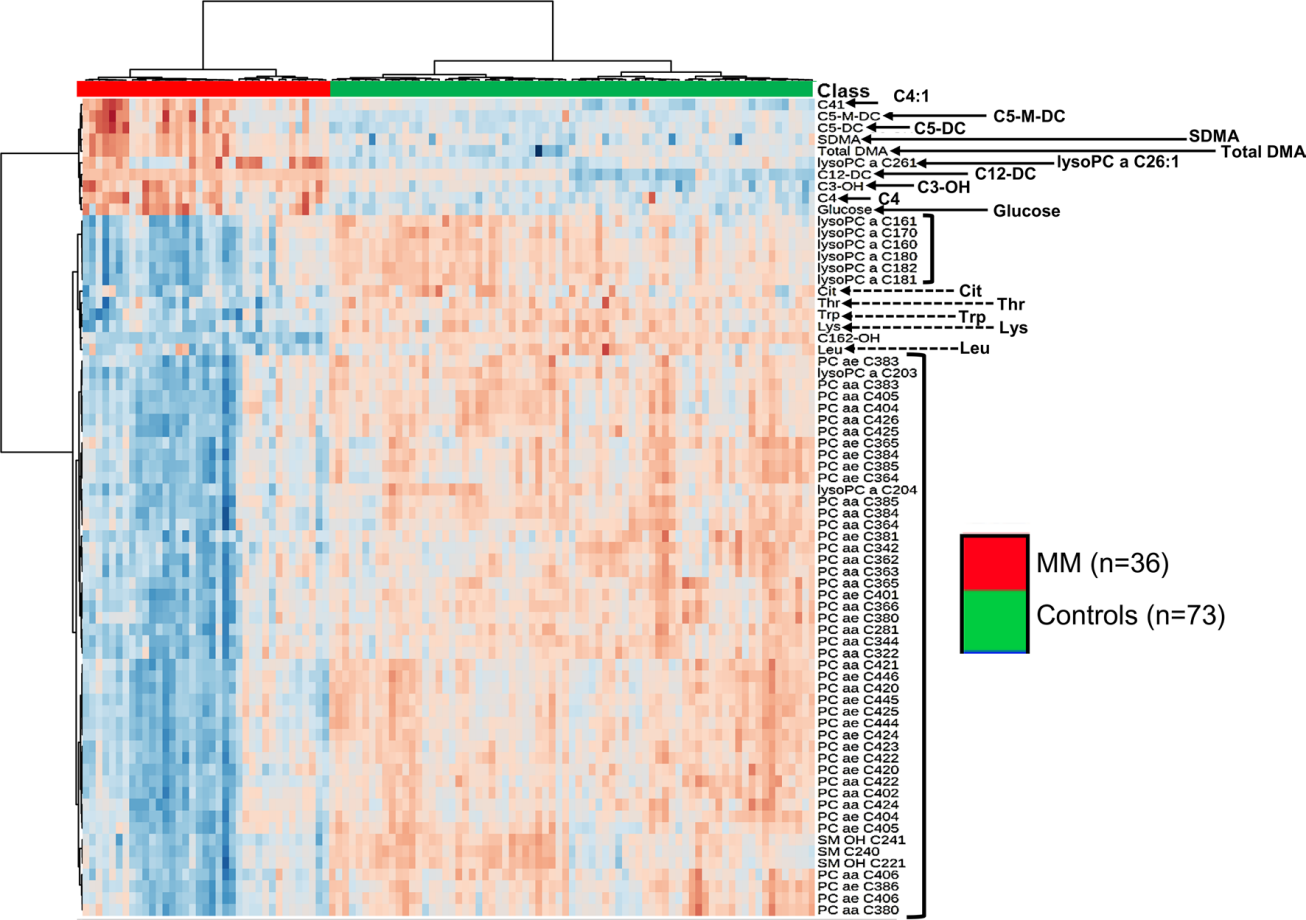
Heatmap of the metabolomics dataset healthy controls (X-axis, green samples) versus MM (X-axis, red samples). In the vertical axis, metabolites are represented. The colors represent the mean concentration of metabolites. They are separated using hierarchical clustering (Ward’s algorithm) with the dendogram being scaled to represent the distance between branches (distance measure: Euclidean) (MetaboAnalyst 3.0. www.metaboanalyst.ca).

**Figure 2:**
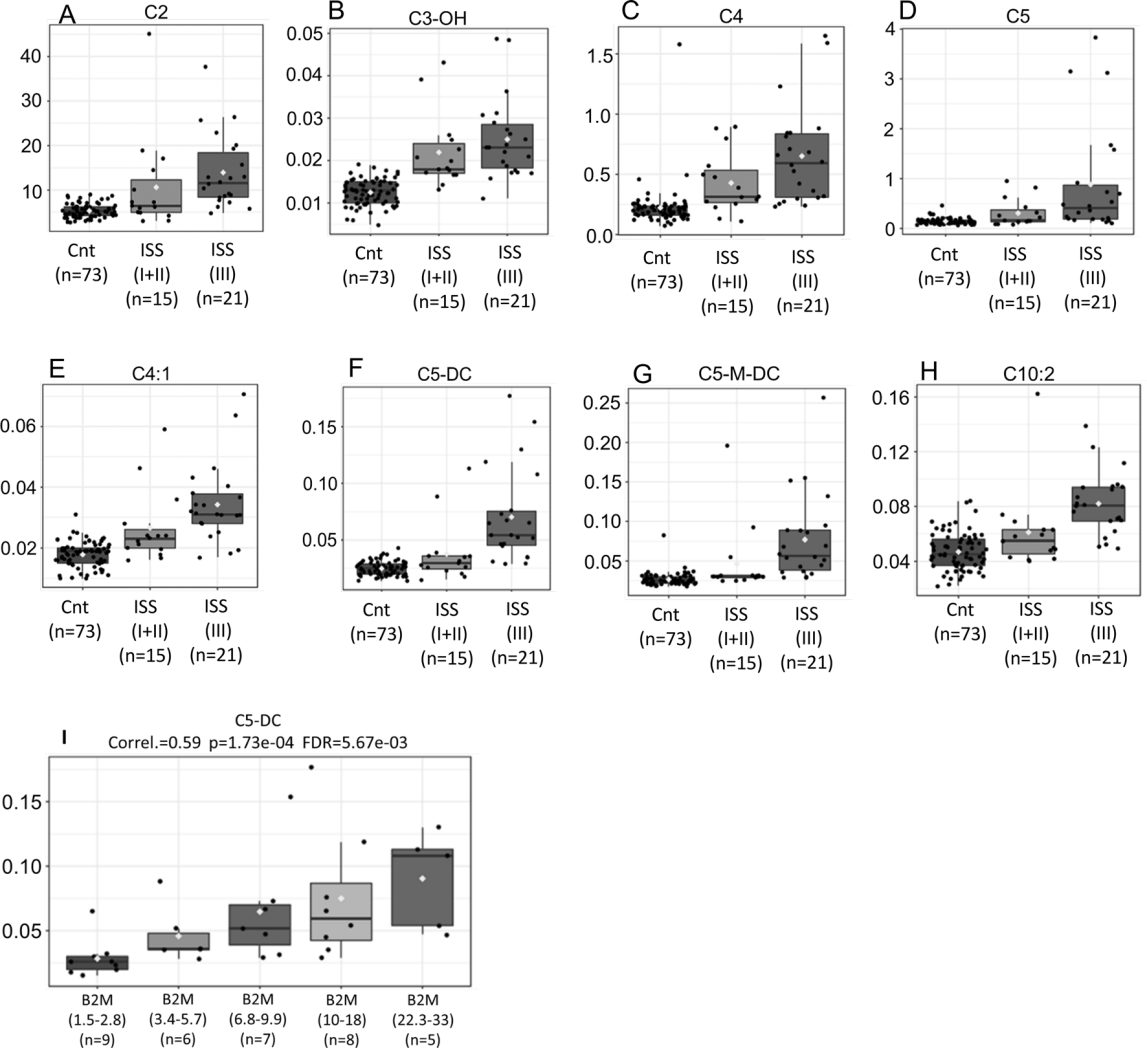
(**A**)–(**G**) and (**H**) Carnitines (Y-axis) levels in controls, MM ISS1 + 2 and ISS 3 (X-axis) showed association with worse prognosis (p values, FDR, and Fisher’s LSD in supplemental material 3). (**I**) Correlations between levels of ß2-microglobulin (X-axis) and plasma concentrations of glutarylcarnitine (C5-DC) (Y-axis). The results show that C5-DC levels correlate with progressive elevations of ß2-microglobulin (Pearson's correlation analysis).

In the present study, 186 metabolites were analyzed in plasma samples with mass spectrometry (targeted) in tandem from controls and MM group. In our initial analysis we did an unsupervised “clustered” assessment using heatmap (Fig. 1, Supplemental material 1), i. e., regardless of our will, the software may or may not be able to find patterns that allow the formation of "clusters". As we can see in Fig. 1, controls and MM groups were clearly discriminated and the 70 most discriminating parameters were depicted in the heatmap. These results reveal that MM samples show elevation of acylcarnitines with short carbon chains (between 2 and 7 carbons) such as C2, C3-OH, C4, C4: 1, C5, C5-DC, C5-M-DC. Our findings are not limited to showing only who is up or down regulated, but more importantly, it shows that these dysfunctions are associated with a worse prognosis (Fig. 2A–H). Reinforcing these findings even further, we carried out Pearson's correlation analysis between increasing levels of ß2-microglobulin (X-axis) and C5-DC (glutarylcarnitine) (Y-axis) (Fig. 2I).The results show correlations of high statistical significance between the plasma concentrations of glutarylcarnitine (C5-DC) that correlate with the progressive elevations of ß2-microglobulin (Fig. 2I).

### Patients with MM have biochemical dysfunctions that suggest a disorder in the metabolism of structural lipids

**Figure 3:**
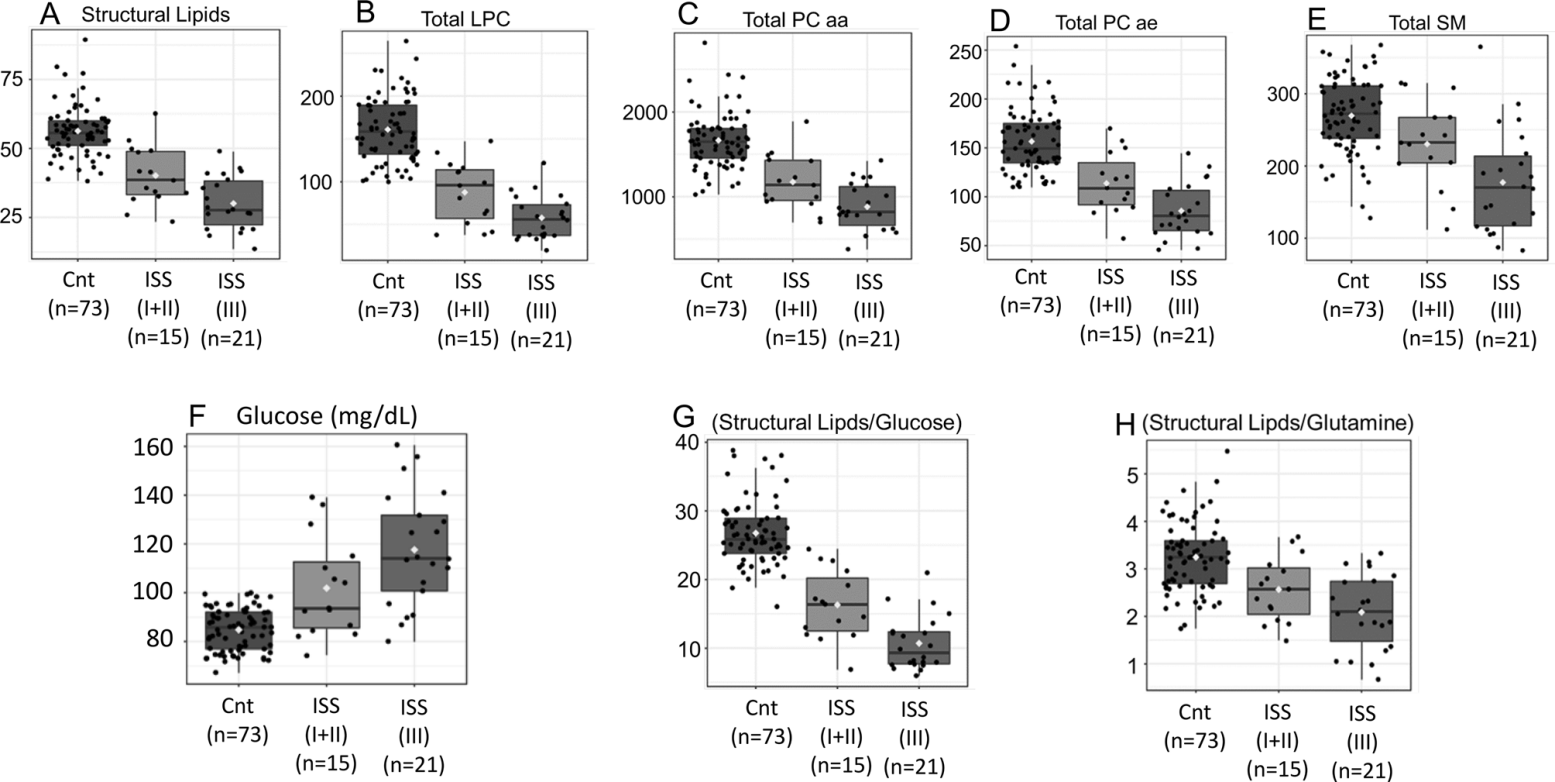
All lipid classes evaluated showed inverse correlation between the levels of the (**A**) sum of all structural lipids (Structural Lipids) (Y-axis), (**B**) lysophosphatidylcholine (Total LPC) (Y-axis), (**C**,**D**) phosphatidylcholine (Total PC aa and Total PC ae) (Y-axis), (**E**) sphingomyelin (Total SM) (Y-axis), and the prognosis of patients with MM. (**F**–**H**) showed significant decrease in the production of glucose and glutamine-dependent lipids (Y-axis) in MM patients in relation to controls (X-axis), a phenomenon that is also accentuated with the increase in ISS (p values, FDR and Fisher’s LSD in supplemental material 3).

The same unsupervised multivariate analysis showed a marked drop in molar concentrations of circulating structural lipids in MM patients. In fact, all lipid classes evaluated show that the lower the levels of sphingomyelin (Total SM), lysophosphatidylcholine (Total LPC), phosphatidylcholine (Total PC aa and Total PC ae) and the sum of all structural lipids (Structural Lipids) the worse the prognosis of patients with MM (Fig. 3A–E).

Considering that the lipids synthesized by humans have glucose and glutamine as main substrates, we also analyzed the proportions between these two metabolites in relation to the sum of all structural lipids.

The results show a biochemical deviation that suggests a significant decrease in the production of glucose and glutamine-dependent lipids, a phenomenon that is also accentuated with the increase in ISS (Fig. 3G,H). Important, glucose levels were directly correlated, not only to M.M. patients but also to increased ISS classification (Fig. 3F).

**Figure 4:**
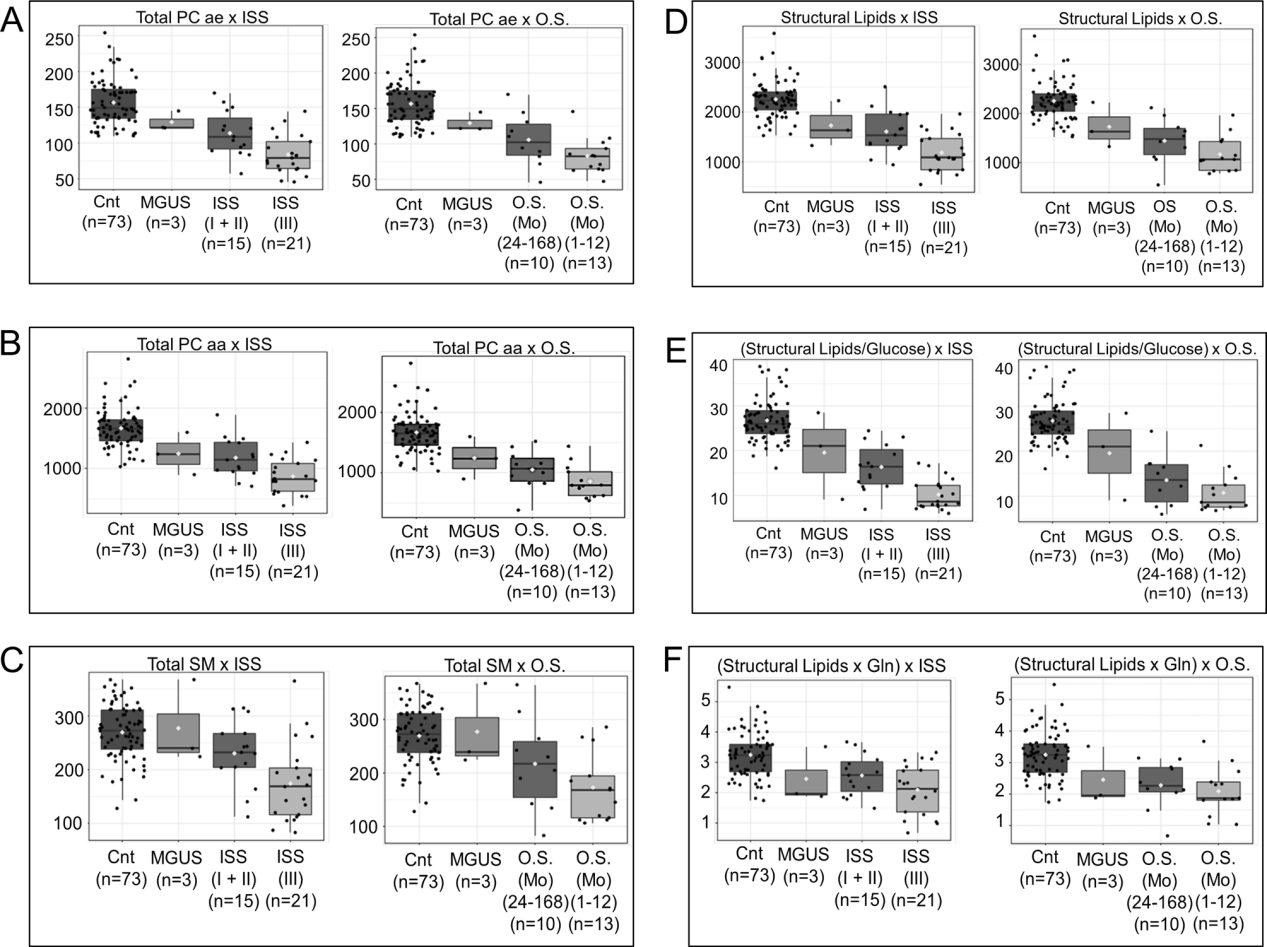
Comparisons among controls, MGUS (n = 3) and MM (n = 36) according to the ISS and the overall survival (OS) (X-axis): (**A**) Total PC aa ) (Y-axis) and (**B**) Total PC ae, phosphatidylcholine) (Y-axis); (**C**) Total SM), sphingomyelin) (Y-axis); (**D**–**F**) Structural lipids and glucose and glutamine-dependent lipids) (Y-axis) (ANOVA and Pearson's Correlation).

Still analyzing the significant drop in structural lipids, we evaluated these levels in premalignant conditions that may precede the onset of MM itself. For this, we added three cases of MGUS beside the controls and MM according to the ISS and the overall survival (OS). The results show that the decrease in structural lipids seen in cases of MGUS, although with values ​​closer to the controls, precedes the emergence of MM and it becomes even smaller as the ISS increases and falls in overall survival, reaching its minimum in those patients whose survival varied from 1 to 12 months (Fig. 4A–F).

### Multiple myeloma is accompanied by a drop in the concentration of essential amino acids and lipids suggesting an absorptive deficit

**Figure 5:**
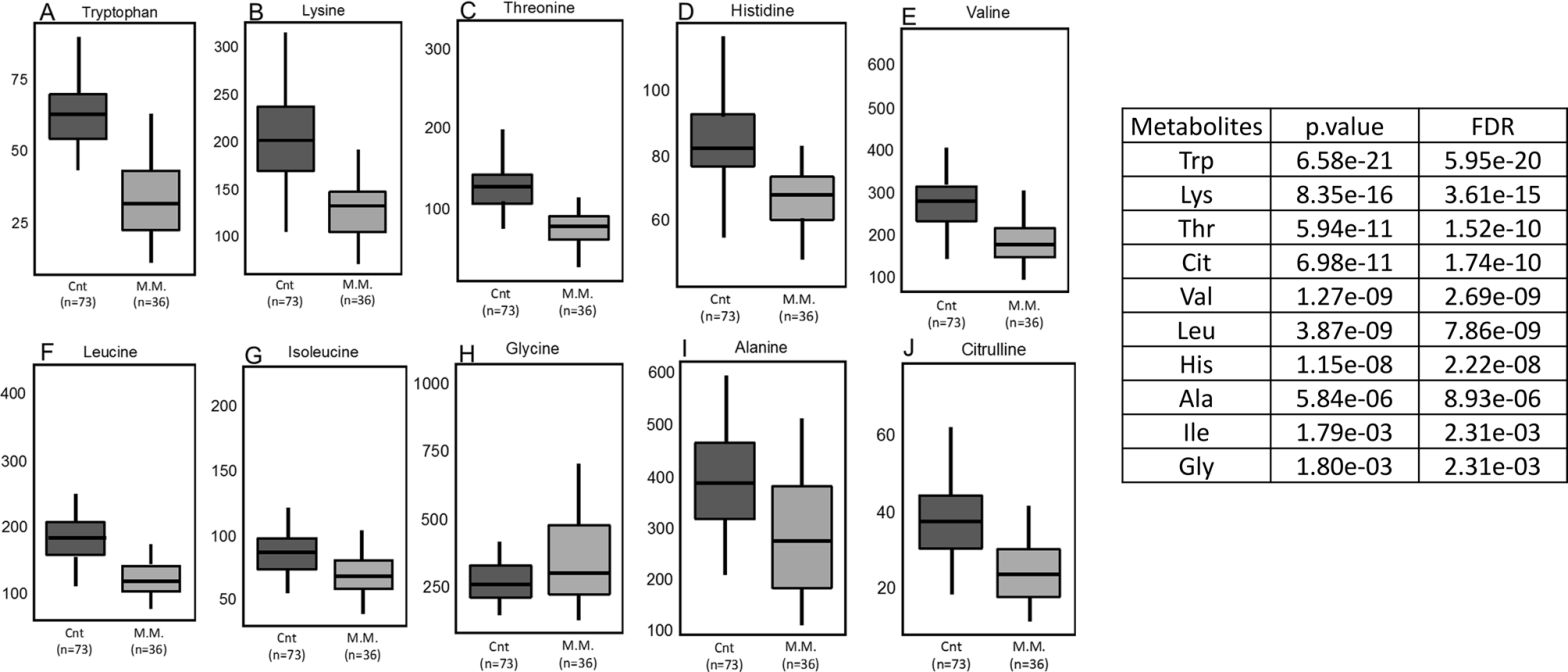
Plasma concentrations (Y-axis) of amino acids (**A**) tryptophan, (**B**) lysine, (**C**) threonine, (**D**) histidine, (**E**) valine, (**F**) leucine, (**G**) isoleucine and (**I**) alanine are significantly decreased in patients with MM compared to controls (X-axis), with only glycine being elevated (**H**). (p values and FDR, t test). (**J**)The concentration of citrulline was significantly decreased in cases of MM when compared to controls (*p* = 6.97e−11, FDR = 1.84e−10, t test).

**Figure 6:**
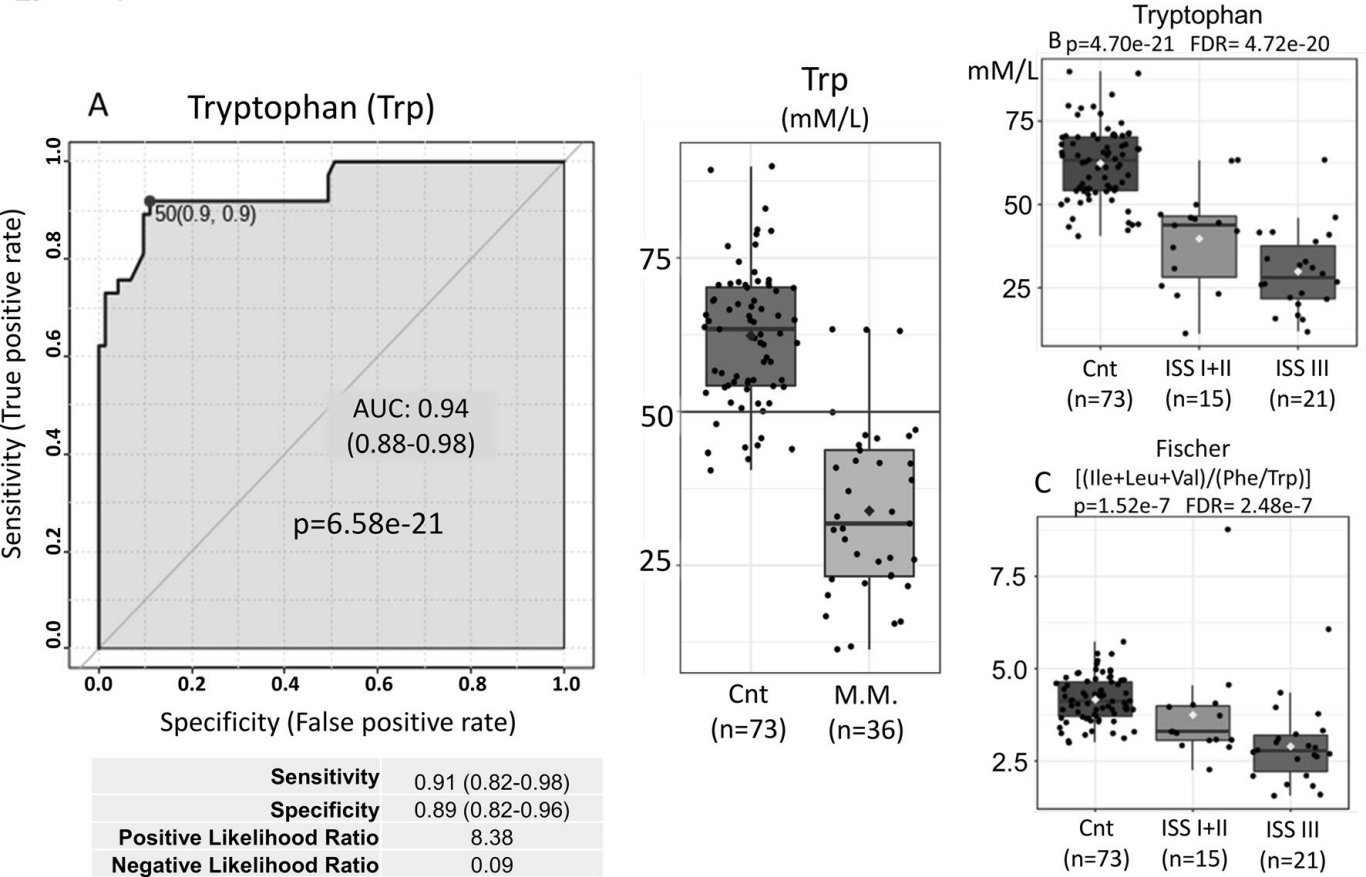
Tryptophan levels (Y-axis) have been able to discriminate MM cases from controls (X-axis) with (**A**) sensitivity of 0.91 (0.82–0.98) (Y-axis), specificity of 0.89 (0.82–0.96) (X-axis), positive likelihood ratio of 8.38 and negative likelihood ratio of 0.09 (AUC 0.947, Roc Curve). (**B**) The separation of MM in ISS (I + II) and (III) demonstrated (X-axis), with statistical significance, that the tryptophan values (Y-axis) are as low as the higher the ISS (*p* = 4.70e−21, ANOVA). (**C**) Ratio [(Ile + leu + Val)/(Tyr + Phe)] (Y-axis) which generates values directly proportional to liver function. The results show that in patients with MM, liver function seems to decrease as the ISS values increase when compared to controls (X-axis) (*p* = 1.52e−7, Fischer test).

Reinforcing the theory of absorptive deficit in MM, the plasma concentrations of several amino acids, especially tryptophan, followed by lysine, threonine, histidine, valine, leucine, isoleucine and alanine (Fig. 5A–I) are significantly decreased in patients with MM compared to controls, with only glycine being elevated (Fig. 5H). Plasma tryptophan concentrations in healthy adults range from 40 to 91 µM/L, which is very close to the control group used here, with an average of 62.34 µM/L (40.5 to 89.9 µM/L).However, in MM patients these values, with an average of 33.86 µM/L (11.3 to 63.4 µM/L), were the ones that fell the most when compared to controls, generating differences of great statistical significance (*p* = 6.58E−21, FDR = 5.95E−20). These differences, by themselves, have already been able to discriminate MM cases from controls with sensitivity of 0.91 (0.82–0.98), specificity of 0.89 (0.82–0.96), positive likelihood ratio of 8.38 and negative likelihood ratio of 0.09 (Fig. 6A).The separation of MM in ISS (I + II) and (III) demonstrated, with statistical significance, that the tryptophan values are as low as the higher the ISS (Fig. 6B).

In an attempt to assess the presence of liver disorders and their eventual association with MM, we used the Fischer ratio [(Ile + leu + Val)/(Tyr + Phe)] which generates values directly proportional to liver function. The results show that in patients with MM, liver function seems to decrease as the ISS values increase when compared to controls (Fig. 6C).

**Figure 7:**
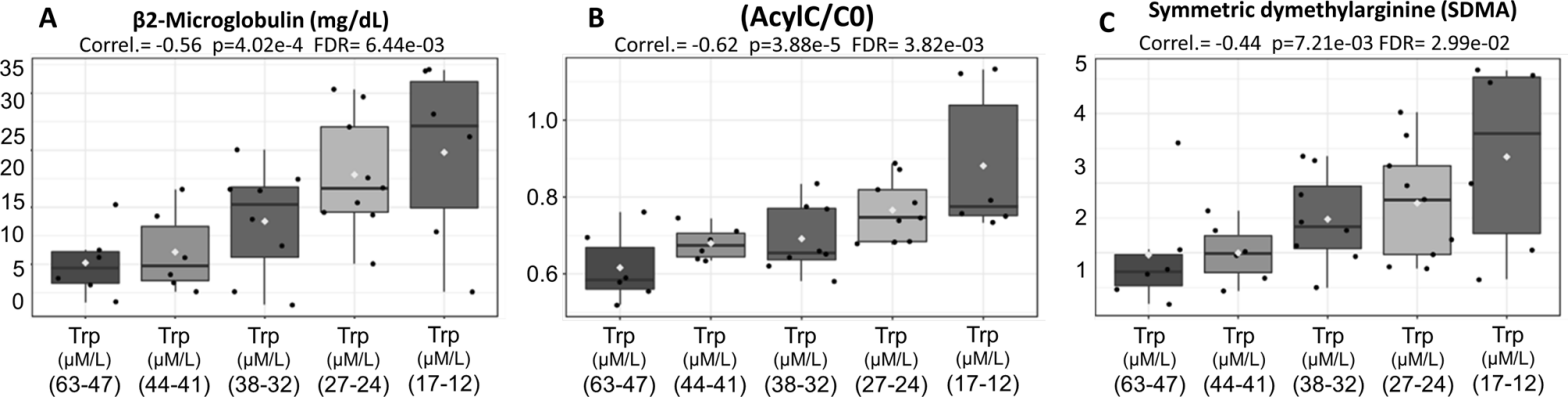
Significant inverse correlation between the progressive drop in tryptophan (X-axis) with the elevation of β2-microglobulin (Y-axis) (**A**)**,** with the accumulation of esterified carnitines in relation to free carnitine (AcylC/C0) (Y-axis) (**B**) and with the increase in systemic methylation levels (Symmetric Arginine Dimethylation, SDMA) (Y-axis) (**C**).

In the present study, we also found a significant inverse correlation between the progressive drop in tryptophan with the elevation of β2-microglobulin (Fig. 7A), with the increase in systemic methylation levels (Symmetric Arginine Dimethylation, SDMA) (Fig. 7B) and with the accumulation of esterified carnitines in relation to free carnitine (AcylC/C0) (Fig. 7C). Tryptophan is also needed to form serotonin, melatonin, kynurenine, and niacin (vitamin B3).

**Figure 8:**
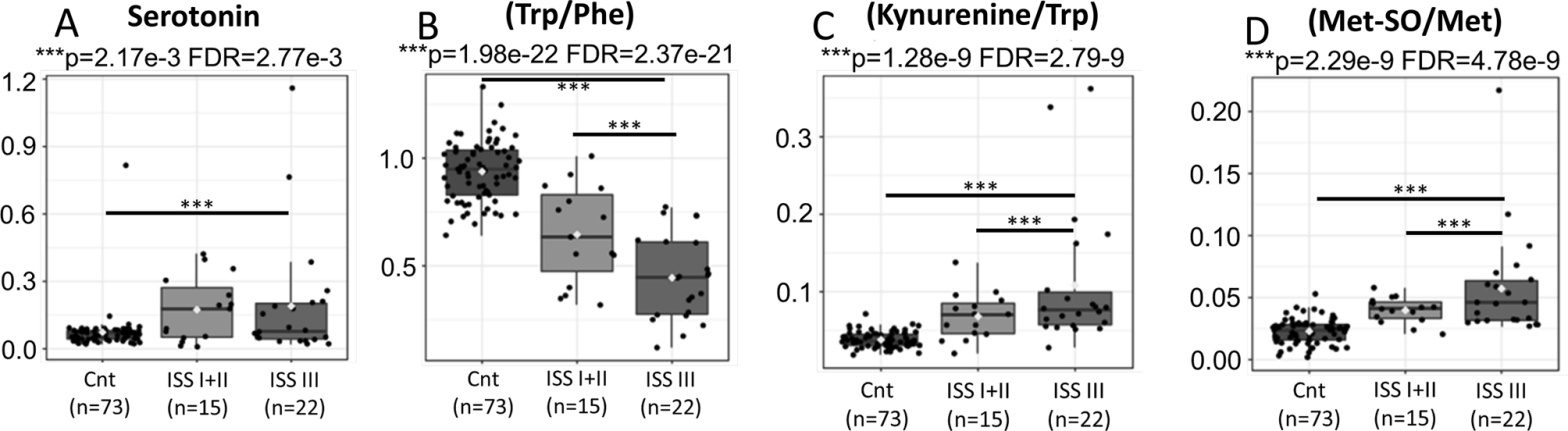
Serotonin (Y-axis) was significantly elevated in cases of MM, without, having a clear association between higher levels and ISS 3 (X-axis) (**A**). Activity of Melatonin Receptor 1 B (Trp/Phe) (Y-axis) shows significant differences between cases and controls, becoming even more evident in ISS 3 cases (X-axis) (**B**). Activity of dioxigenases (kynurenine/tryptophan) (Y-axis) (**C**) and oxidative stress (Met-SO/Met) (Y-axis) (**D**) demonstrate that, in addition to being significantly elevated in the cases of MM, they are even higher in the cases classified as ISS 3 (X-axis).

In our series, serotonin was significantly elevated in cases of MM, without, however, having a clear association between higher levels and ISS3 (Fig. 8A). With the intention of evaluating in more detail the possible effect of this pathway in cases of MM, we compared, from the perspective of the activity of Melatonin Receptor 1 B (Trp/Phe), the controls to MM cases divided according to ISS.

The results show significant differences between cases and controls, becoming even more evident in ISS3 cases (Fig. 8B). From the perspective of the activity of dioxigenases (kynurenine/tryptophan) our results demonstrate that, in addition to being significantly elevated in the cases of MM, the activity of dioxigenases is even higher in the cases classified as ISS 3 (Fig. 8C).

The concentration of citrulline (Fig. 5J), a non-proteinogenic amino acid whose plasma concentration normally ranges from 16 to 51 µM/L, was significantly decreased (*p* = 6.97e−11, FDR = 1.84e−10), with average of 24.03 µM/L (11.30–41.60 µM/L), in cases of MM when compared to controls, with average of 38.53 µM/L (17.90–76 µM/L).

**Figure 9:**
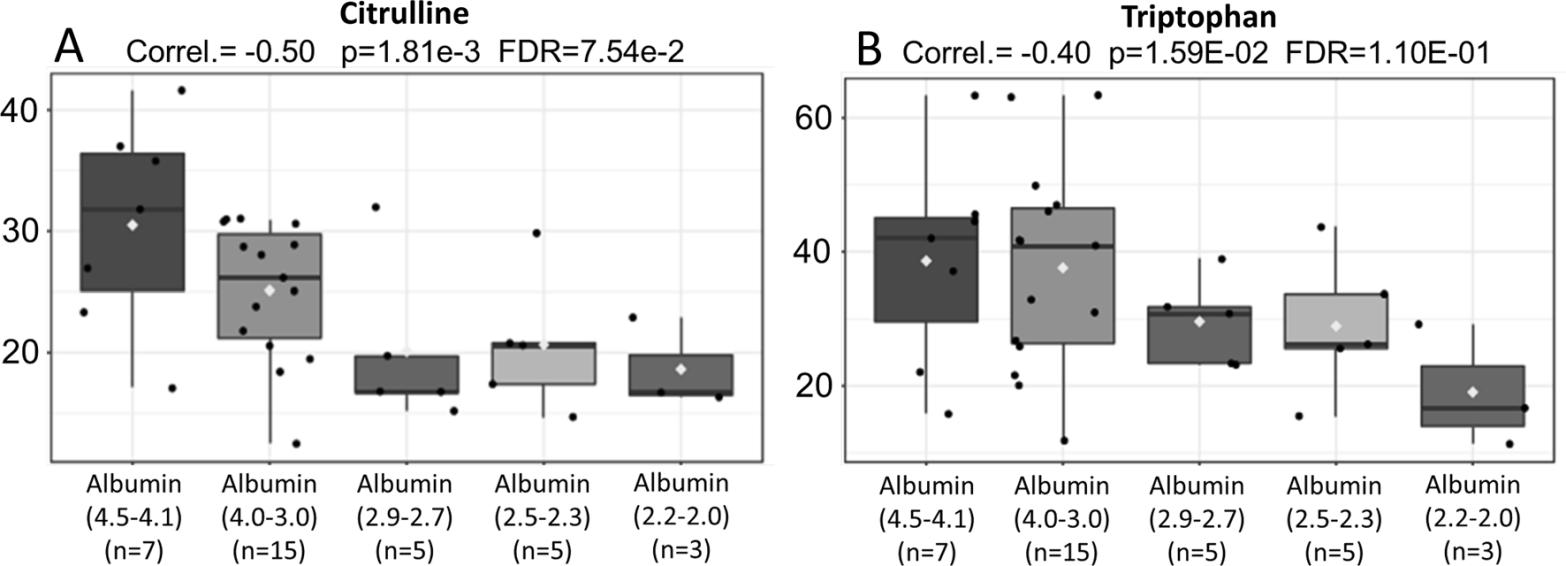
Pearson's correlation shows that the concentration of citrulline (**A**) and tryptophan (**B**) (Y-axis) decreases, in parallel, with the fall of albumin (X-axis).

Reinforcing the possibility of the occurrence of absorptive/nutritional disorders in MM we evaluated, which metabolites correlate with the albumin drop. The results show that the concentration of citrulline and tryptophan (Y-axis) decreases, in parallel, with the fall of albumin (X-axis) (Fig. 9A,B).

### Metabolite Set Enrichment Analysis (MSEA) reinforce aminoacidopathies as biochemical disturbances underlying multiple myeloma

Results from MM treatment-naive patients exhibit biochemical features highly similar to the Inborn Errors of Metabolism such as Hartnup Disease (*p* = 2.06e−34 and FDR = 1.85e−33), but also Fatty Acids Oxidation Defects (FAOD) (Supplemental material 2). Important, when interrogated for metabolic deviations, MSEA results revealed, in support of the Hartnup-like theory, a significant enrichment of metabolites related to Tryptophan Metabolism where identified at elevated significance (*p* = 2.89e−85 and FDR = 8.39e−84) (Supplemental material 2).

These types of energy generation deficiencies, more frequently located in mitochondria, are biochemically characterized by built-ups, in blood and tissues, of esterified short, medium, long, and/or very-long acylcarnitine types.

Indeed, ANOVA analysis of the proportions between the molar sum of all acylcarnitines species (AcylC) in relation to molar values of unconjugated free carnitine (C0) revealed values at least 3 times higher in MM patients exhibiting lower tryptophan values (*p* = 3.88e−5, FDR = 3.82e−3) (Fig. 7B). Of note, the normal population values generated by this ratio (AcylC/C0) are usually < 0.4.

From the perspective of excessive free radical production that usually appears as a result of mitochondrial dysfunctions, our results detected significant elevations of oxidative stress in MM patients by measuring the proportions between the systemic values of methionine sulfoxyde (Met-SO) to unmodified methionine (Met). Indeed, results were directly correlated to increases in both ISS and percentage of cancer cells (Fig. 8D).

## Discussion

Recent studies show that metabolism plays an important role in metabolic reprogramming, not only in cancer, but also in events related to aging, senescence, and development^17^. Therefore, metabolomics has been used in diagnosis, pathogenesis, disease progression, biomarkers identification, and to provide new insights into the aspects of tumor biology, collaborating to new therapeutic proposals in cancer^18–20^.

Some recent works have pointed metabolic pathways and possible new therapeutic targets in MM^14,15,21,22^. Also, Ludwig et al.^23^ identified that the majority of the most represented metabolites in MGUS and MM are lipids. However, most of the metabolic changes occurred in MGUS (200 annotated metabolites compared to controls, 50%) been the minor part of the metabolic changes occurred in MM (26 metabolites annotated, < 3%), suggesting that robust metabolic changes were present in the pre-malignant phase of disease.

**Figure 10:**
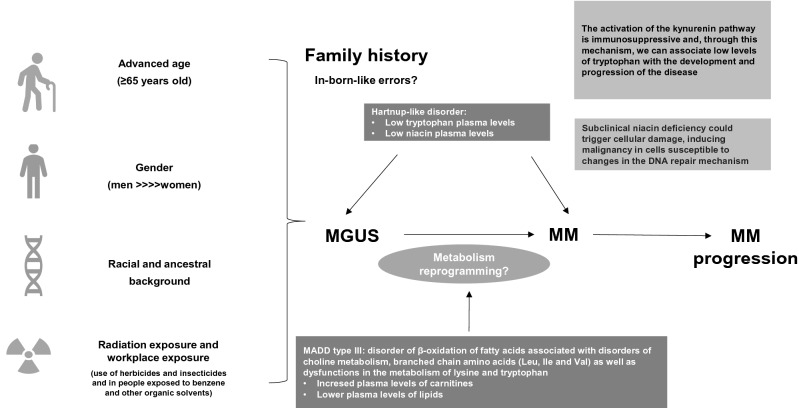
Inborn-like errors as risk factors to MM development and/or disease progression.

In this sense, the present study aimed to evaluate the plasma metabolomic profile of patients diagnosed with MM, focusing on the profile evaluation of different disease stages in relation to healthy/non-oncologic patients. Our study showed that MM patients at diagnosis present a disorder of mitochondrial complexes I and II and a Hartnup-like disturbance as underlying conditions, also influencing different stages of the disease. Therefore, an emerging question is: are inborn-like errors risk factors to MM development and/or contribute to disease progression (Fig. 10).

Thanks to different acylcarnitine constituents and variable number of carbon atoms, it is possible to pinpoint the dysfunctional acyl-Coenzyme A dehydrogenase enzyme(s) due to their relative specificity towards species containing particular number of carbon atoms.

Our results showed an increase in short, medium, and long-chain species of acylcarnitines in plasma metabolomic profile of MM and its respective ISS stages. Similar results have been reported for MGUS, newly diagnosed MM patients, and relapsed patients^14^. Acylcarnitines are a class of metabolites that play an important role in the transport of fatty acids into mitochondria for subsequent β-oxidation, providing energy to cells^24^. As such, carnitine metabolism is involved with intracellular sugar and lipid metabolism^25^, as well as fatty acid oxidation and ketone body production^26^.

The metabolic picture found in the present study, is very suggestive of an innate metabolism error called Multiple Acyl-CoA Dehydrogenase Deficiency (MADD)^27^, also known as glutaric aciduria type II. In this condition, there is an increase in both short and medium/long-chain acylcarnitines due to the global deficiency of important acylCoa-dehydrogenases during oxidative phosphorylation (OXPHOS)^27^. More specifically, this is a disorder of Mitochondrial Complexes I and II inherited in an autosomal recessive manner, characterized by disturbances in Electron Transfer Flavoprotein (ETF), ETF-ubiquinone Oxidoreductase (ETF-QO) or by dysfunctions in the metabolism and transport of Flavin^28^.

It is, therefore, a disorder of β-oxidation of fatty acids that can be accompanied by biochemical disorders of choline, branched chain amino acids (Leu, Ile, and Val) as well as lysine and tryptophan metabolisms. The situation we have here would fit better in MADD type III, which is characterized by the appearance of symptoms in adult life with intensities ranging from mild or absent up to cases of severe metabolic crises^29^. Common biochemical dysfunctions include elevations in transaminases and ammonia suggesting liver dysfunctions related to the urea cycle. Metabolic acidosis, non-ketotypic hypoglycemia, generalized weakness, cardiac and muscle disorders with elevated creatine phosphokinases are also common^27^.

Recent findings, in support of impairments located in complexes I and II, was the evidence that the activity of the electron transport chain seems to be a predictor of response in MM patients^30^. However, before assuming that these are, in fact, dysfunctions in mitochondrial complexes I and II, it is important to note that, unlike MADD, where hypoglycemia is a frequent finding, in the cases of MM studied here we observed a significant increase in plasma glucose levels (Fig. 3F). In fact, another situation that can mimic MADD, in several aspects, is riboflavin (B2 vitamin) deficiency, where hyperglycemia is a common feature. In addition, B2 vitamin deficiency is also found in other chronic diseases such as rheumatic fever, tuberculosis, bacterial endocarditis, thyroid and liver dysfunction (https://www.sciencedirect.com/topics/medicine-and-dentistry/riboflavin-deficiency/pdf.)

Low levels of riboflavin mimic MADD since this vitamin is the precursor molecule of FAD and FMN, both used in the oxidative phosphorylation process. Therefore, its vitamin low level can interfere with the correct functioning of acylCoA dehydrogenases, leading to dysfunctions in the mitochondria complexes I and II. On the other hand, dysfunctions in the riboflavin transporters (SLC52A1 (RFT1), SLC52A2 (RFT3) and SLC52A3 (RFT2) can lead to riboflavin deficiency which results in biochemical and clinical abnormalities that resemble those seen in MADD (https://www.sciencedirect.com/topics/medicine-and-dentistry/riboflavin-deficiency/pdf)^28,31^. Riboflavin deficiency rarely occurs isolated, but usually, in combination with decrease in other water-soluble vitamins, which may suggest the presence of absorptive dysfunctions. Malabsorption can happen as a result of gastro-intestinal diseases, such as celiac disease, diarrhea, enteritis, biliary atresia, and irritable bowel syndrome (https://www.sciencedirect.com/topics/medicine-and-dentistry/riboflavin-deficiency/pdf).

Other possibility, which does not exclude the previous hypothesis for elevated levels of carnitines in MM group, could be a metabolic reprogramming to regulate carnitine synthesis levels, associated with other metabolic pathways, such as lipids, as a source of metabolites for rapid cell proliferation and disease progression^24,32–35^.

Our results also showed a significant change in plasma phosphatidylcholine (PC) with lower concentrations in the MM patients and respective ISS groups. Lipids are biomolecules that have a structural and functional role in cells^36^. Lipid metabolism participates in the regulation of many cell processes, such as cell growth, proliferation, differentiation, survival, apoptosis, inflammation, motility, membrane homeostasis, chemotherapy response and drug resistance^37^. PC is the most abundant glycerophospholipid in most eukaryotic membranes, and PC and LPC comprise 60–70% and 10–20% of human plasma circulation, respectively^38^. A recent work describing lipidomic results in purified plasma cells collected from MM patients showed reduced levels of PC in MM compared with healthy plasma cells^39^.

LPC is formed by the hydrolysis of PC by the phospholipase A2 enzyme^40^. LPC is described as an important low-density protein (LDL)/bioactive lipid and is responsible for the inflammatory effects of oxidized LDL on endothelial cells and stimulates basic fibroblast growth factor (bFGF) and the granulocyte–macrophage–cytokine colony-stimulating factor (GM-CSF)^40^. LPC have previously been reported as a biomolecule involved in inflammatory stimuli, promoting the release of IL-6 and other inflammatory factors, and resulting in the progression of MM^21^. Our LPC analysis showed lower plasma levels in controls versus MM and the decrease is more evident in advanced stages ISS 3. Thus, our results suggest that LPC species are involved in the inflammatory state of MM.

Sphingolipids (SLs) are another family of lipids that have a structural role in the plasma membrane, and the products of their metabolism (ceramides, ceramide-1-phosphate, sphingosine, sphingosine-1-phosphate) play important roles as bioactive lipids^37^. Sphingolipid molecules are highly regulated by metabolic enzymes, which altered expression or activity play important roles in inducing cell death or survival^41^. Here, SM presented significantly decreased levels in plasma. These results suggest that SM hydrolysis can be part of the systemic metabolic regulation/reprogramming of MM.

Additionally, our results show that the decrease in structural lipids seen in cases of MGUS, although with values closer to the controls, precedes the emergence of MM and it becomes even smaller as the ISS increases, reaching its minimum values in those patients whose survival varied from 1 to 12 months. Thus, these findings suggest a causal relationship between the increase in biochemical deviation, not only with the onset of malignancy, but also with the increase of ISS score, i.e., MM prognosis.

The biochemical process that allows the absorption of tryptophan in the intestine is the same used in the absorption of several other amino acids, especially the neutral amino acids. The decrease in tryptophan absorption may be secondary to mutations in the SLC6A19 gene, that encodes a sodium-dependent carrier protein responsible for the absorption of amino acids in the intestine and reabsorption in the kidneys, characterizing the Hartnup's disease^42^.That is the biochemical scenario described in Fig. 5, in fact, highly suggestive of this condition, since there is a drop in the absorption of tryptophan, with consequent decrease in the absorption of several neutral amino acids such as lysine, threonine, histidine, valine, leucine, isoleucine and alanine, with the exception of glycine (Supplemental material 2).

These results also demonstrate that, much more than the simple association, the progressive drop in tryptophan levels is closely followed by biochemical dysfunctions related to a worse prognosis in an inversely proportional way^43^. Drop in tryptophan seems also to constitute a biochemical dysfunction capable of negatively interfering with mitochondrial functioning, which can be seen by the progressive and significant accumulation of acylcarnitines as the tryptophan molar concentrations decrease (Fig. 7B). Currently it is not possible to access blood samples 20 years or more before the onset of MM to prove that there has been already a metabolic alteration that may predispose to the origin of the neoplasm. One possibility would be to monitor patients with Hartnup disease and see if they develop lymphoproliferative diseases. This theory would also be applied to chronic lymphocytic leukemia and indolent lymphomas, for which there is no known etiologic agent.

Recent studies point serotonin as a tumor growth factor (carcinomas, gliomas, and carcinoids), associated with cancer cell migration, metastasis, and tumor angiogenesis^44^. However, the signaling pathways associated with serotonin (and other monoamines) for tumor progression are still poorly understood. Regarding serotonin, it is important to mention that, apparently, the inhibition of its receptors is followed by an anti-myeloma effect mediated by apoptosis^45^. In our series, serotonin was significantly elevated in cases of MM, without, however, having a clear association between higher levels and ISS3. Regarding melatonin and its circadian rhythm controlling activity, recent studies have detected significant associations between disturbances in circadian rhythm and increased risk of developing MM^46^.With the intention of evaluating in more detail the possible effect of this pathway in cases of MM, we compared, from the perspective of the activity of Melatonin Receptor 1 B (Trp/Phe), the controls to MM cases divided according to ISS. The results show significant differences between cases and controls, becoming even more evident in ISS3 cases.

In Hartnup disease, reduced intestinal absorption of essential amino acids, such as tryptophan, and increased tryptophan loss in the urine lead to reduced availability of tryptophan for the synthesis of niacin as well as profound immunological repercussions due to disturbances in indoloxigenases activity. Niacin, through its two coenzyme forms, niacinamide adenine dinucleotide (NAD+, NADH+H+) and niacinamide adenine dinucleotide phosphate (NADP+, NADPH+H+) act in a number of redox reactions spanning from energy production (NAD+ and NADH) to lipid biosynthesis and oxidative stress control (NADPH). It is still important to note that a variety of proteins associated with DNA repair undergo post-translational modifications such as adenosine diphosphate (ADP)-ribosylation and one of the characteristics of niacin deficiency is the increase in the breaking of the strands of DNA, whose repair does not occur promptly^47,48^. In this scenario, it has been proposed that subclinical niacin deficiency could trigger cellular damage, inducing malignancy in cells susceptible to changes in the DNA repair mechanism ^47,48^, and being a possible link to clarify what is the relationship between the innate error and the genetic-molecular changes that gave rise to the tumor clone in MGUS.

The production process of kynurenine is catalyzed by the enzymes tryptophan and indolamine 2,3-dioxigenases (IDO). Its dysfunctions are directly involved, not only in the suppression of T lymphocyte activity, but are also associated with disorders in lipid metabolism, hepatic steatosis, glutaric acidemia and vitamin B6 deficiencies^49^. More recent studies show a prognostic role of this metabolic pathway in the precursor lesions of hematological cancers^50^.

In order to assess the possible effect of this pathway in MM cases, we compared the controls to MM cases divided according to the ISS, from the perspective of the activity of dioxigenases (kynurenine/tryptophan). The results demonstrate that, in addition of being significantly elevated in the cases of MM, the activity of dioxigenases are even higher in the cases classified as ISS 3. Therefore, the activation of the kynurenin pathway is immunosuppressive and, through this mechanism, we can associate low levels of tryptophan with the development and progression of the disease.

The concentration of citrulline was significantly decreased in cases of MM when compared to controls. Considering that citrulline metabolism occurs, to a large extent, at the level of the small intestine, the measurement of citrulline concentration is directly related to the mass of functional enterocytes which, when low, is associated with the presence of absorption disorders^51–53^. Supporting this finding, low levels of citrulline were found in patients with hematological malignant and premalignant conditions, it is also associated with an increased risk of graft versus host disease (GVHD) Rashidi et al.^54^. Reinforcing the possibility of the occurrence of absorptive/nutritional disorders in MM, we evaluated which metabolites correlate with the albumin drop. The results show that the concentration of citrulline and tryptophan decreases, in parallel, with the fall of albumin proving the correlation between the functional integrity of the small intestine and its influence on variables that routinely are used in the MM ISS risk classification.

Although the present work had a limitation in the number of the samples of patients diagnosed in the ISS I group, it did not avoid deep metabolomic analyses. Other possible limitation of this study is the absence of other MM risk-group markers, such as fluorescence in situ hybridization profile, to define prognosis of our patients and compare them with metabolic findings.

## Conclusion

In conclusion, this work identified significant biochemical disturbances occurring in the metabolism of amino acids and derivatives, acylcarnitines, as well as in structural lipids biosynthesis in blood of patients with MM and we purpose that inborn-like errors of metabolism can be an underlying risk factor for MM development (Fig. 10). Our results also showed relevant metabolic changes during MM progression from ISS 1 to 3 pointing the importance of tryptophan and kynurenine pathway as possible targets in MM treatment.

## Material and methods

### Ethical aspects

This study was approved by the Research Ethics Committee of the Universidade Federal de São Paulo—UNIFESP (#1123/2018). All patients and healthy controls provided written informed consent prior of plasma processing. We confirm that all the experimental protocols were in accordance with guidelines of national/institutional or Declaration of Helsinki.

### Nested case control setting

MM patients included in the present study were diagnosed according to the International Myeloma Working Group (IMWG) criteria^55^ and were treated at the São Paulo Hospital, Federal University of São Paulo, located in São Paulo, SP, Brazil. The International Staging System (ISS) was used to classify disease progression^8^. Patients’ characteristics and clinical data are shown in Table 1. Patients were classified as ISS 1 (N = 5), ISS 2 (N = 10) and ISS 3 (n = 21). For most of the analyzed metabolites, ISS 1 and 2 behave in a similar way, with more relevant difference noticed in ISS 3 patients. Therefore, to improve statistical analyses, ISS 1 and 2 were analyzed together, as one group. Control group was composed by 73 volunteers (female n = 32/male n = 41) (median age = 62 years) with available, prospectively collected, samples during the execution of the São Paulo Population Public Health Research Project (ISA 2008) designed to prospectively analyze the use of Public Health Service in the city of São Paulo, SP, Brazil, by the Public Health School at the State University of São Paulo.

**Table 1:** Clinical data of MGUS and MM patients (according to ISS).

MGUS	MM	ISS I		ISS II		ISS III	
		n = 5	%	n = 10	%	n = 21	%
59 (43–72)	Age (median, range), years	54 (40–69)		59 (28–76)		64 (43–80)	
Gender							
1	Female	3	60.0	3	30	11	52.4
2	Male	2	40.0	7	70	10	47.6
Immunoglobulin							
2	IgG	2	40.5	8	80	7	33.3
0	IgA	1	10.0	1	10	11	52.4
0	IgM	0	0	0	0	0	0
0	Non-IgG/IgA/IgM	2	40.5	1	10	3	14.3
	Light chain						
3	λ	2	40.0	4	40	8	38.0
0	κ	3	60.0	6	60	13	62.0
Durie–Salmon Staging System							
	IA	0	0	1	10	0	0
	IB	0	0	0	0	0	0
	IIA	0	0	1	10	0	0
	IIB	0	0	0	0	0	0
	IIIA	5	100	5	50	11	52.4
	IIIB	0	0	3	30	10	47.6

*MGUS* Monoclonal gammopathy of undetermined significance, *MM* Multiple myeloma, *ISS* International Staging System.

All plasma samples were collected in EDTA-containing tubes, centrifuged at 1000-x g for 10 min, transferred to a new tube and stored immediately at − 80 °C until analysis.

### Metabolomic analysis

Plasma samples stored at − 80 °C were kept on dry ice during transportation. Absolute quantification (µM/L) of peripheral blood metabolites was achieved by targeted quantitative profiling of 186 annotated metabolites by electrospray ionization (ESI) tandem mass spectrometry (MS/MS) in plasma samples, using SCIEX 5500 QTRAP (SCIEX, Darmstadt, German), blinded to any phenotype information, on a centralized, independent, fee-for-service basis at the quantitative metabolomics platform from BIOCRATES Life Sciences AG, Innsbruck, Austria (https://biocrates.com/).

The experimental metabolomics measurement technique is described in detail by patent US 2007/0004044 (accessible online at https://www.freepatentsonline.com/20070004044.html). Briefly, a targeted profiling scheme was used to quantitatively screen for fully annotated metabolites using multiple reaction monitoring, neutral loss, and precursor ion scans. Quantification of metabolite concentrations and quality control assessment was performed with the MetIQ software package (BIOCRATES Life Sciences AG, Innsbruck, Austria) in conformance with 21CFR (Code of Federal Regulations) Part 11, which implies proof of reproducibility within a given error range. An xls file was then generated, which contained sample identification and 186 metabolite names and concentrations with the unit of μmol/L of plasma (https://biocrates.com/).

In total, 186 annotated metabolites were quantified using the p180 kit (BIOCRATES Life Sciences AG, Innsbruck, Austria), being 40 acylcanitines (ACs), 21 amino acids (AAs), 19 biogenic amines (BA), sum of hexoses (Hex), 76 phosphatidylcholines (PCs), 14 lyso-phosphatidylcholines (LPCs) and 15 sphingomyelins (SMs). Glycerophospholipids were further differentiated with respect to the presence of ester (a) and ether (e) bonds in the glycerol moiety, where two letters denote that two glycerol positions are bound to a fatty acid residue (aa = diacyl, ae = acyl-alkyl), while a single letter indicates the presence of a single fatty acid residue (a = acyl or e = alkyl) (https://biocrates.com/).

For metabolomic data analysis, log-transformation was applied to all quantified metabolites to normalize the concentration distributions and uploaded into the web-based analytical pipelines MetaboAnalyst 3.0 (www.metaboanalyst.ca) and Receiver Operating Characteristic Curve Explorer & Tester (ROCCET) available at https://www.roccet.ca/ROCCET for the generation of uni and multivariate Receiver Operating Characteristic (ROC) curves obtained through Support Vector Machine (SVM), Partial Least Squares-Discriminant Analysis (PLS-DA) and Random Forests as well as Logistic Regression Models to calculate Odds Ratios of specific metabolites (https://www.roccet.ca/ROCCET). ROC curves were generated by Monte-Carlo Cross Validation (MCCV) using balanced sub-sampling where two thirds (2/3) of the samples were used to evaluate the feature importance. Significant features were then used to build classification models, which were validated on the 1/3 of the samples that were left out on the first analysis. The same procedure was repeated 10–100 times to calculate the performance and confidence interval of each model. To further validate the statistical significance of each model, ROC calculations included bootstrap 95% confidence intervals for the desired model specificity as well as accuracy after 1000 permutations and false discovery rates (FDR) calculation (https://www.roccet.ca/ROCCET).

In addition to individual metabolite quantification, groups of metabolites related to specific functions were assembled as ratios based on previous observation that the proportions between metabolite concentrations can strengthen the association signal and at the same time provide new information about possible metabolic pathways^56–58^.

Groups of lipids, important to evaluate lipid metabolism, were also analyzed by summing: 1. Total lysophosphatidylcholines (total LPC), 2. Total acyl-acyl and 3. Total acyl-alkyl phosphatidylcholines (total PC aa and total PC ae, respectively), 4. Total sphingomyelins (total SM, 5. Sum of total (LPC + PC aa + PC ae + SM) lipids (Structural lipids), 6; Total lipids/glucose and total lipids/glutamine.

Biochemical indicators of liver metabolism and function were obtained by applying either the classical (leucine + isoleucine + valine/(tyrosine + phenylalanine) or variations (Val/Phe, Xleu/Phe) of the Fischer’s quotient.

Metabolic indicators of isovaleric acidemia, tyrosinemia, urea cycle deficiency and disorders of ß-oxidation were calculated by adopting the ratios of valerylcarnitine to butyrylcarnitine (C5/C4), tyrosine to serine (Tyr/Ser), glycine to alanine and glutamine (Gly/Ala, Gly/Gln)), respectively^59,60^.

Proxies for enzyme function related to the diagnosis of very long-chain acyl-CoA dehydrogenase (VLCAD) and type 2 carnitine-palmitoyl transferase (CPT-2) deficiencies were achieved by assembling the ratios (C16 + C18:1/C2), (C14:1/C4), (C14:1-OH/C9), (C14/C9) and (C14:1/C9)^57,61–66^.

Levels of methionine sulfoxide (Met-SO) alone or in combination to unmodified methionine (Met-SO/Met) as well as symmetric (SDMA), asymmetric (ADMA) and total dimethylation of arginine residues (Total DMA) were quantified to gain access to ROS-mediated protein modifications as well as to systemic arginine methylation status, respectively.

Additionally, groups of AAs were computed by summing the levels of amino acids (AA) belonging to certain families or chemical structures depending on their functions such as the sum of: 1. branched-chain (Leu + Ile + Val) amino acids (BCAA), 2 tryptophan/phenylalanine ratio (Trp/Phe)^56^ 3. levels of methionine sulphoxide (Met-SO) alone or in 4. combination to unmodified methionine (Met-SO/Met) as well as 5. symmetric (SDMA), 6. asymmetric (ADMA) and 7. total dimethylation of arginine residues (Total DMA) were quantified to gain access to insulin resistance, activity of the Melatonin Receptor 1B (MTNR1B)^56^, ROS-mediated protein modifications as well as to systemic arginine methylation status respectively^67^.

### Metabolite set enrichment analysis (MSEA)

Data generated through targeted quantitative electrospray ionization tandem mass spectrometry (ESI–MS/MS) was uploaded to the Metabolite Set Enrichment Analysis (MSEA), available at www.metaboanalyst.ca as an “in silico” unsupervised tool to aid in the identification of biochemical disturbances present in MM patients. All the metabolic data of cases and controls are deposited in MetaboLights platform (MTBLS 1808).

## Supplementary information

Supplementary Table Legends.

Supplementary Tables.

## References

[1] Palumbo A, Anderson K (2011). Multiple myeloma. N. Engl. J. Med..

[2] Bringle K, Rogers B (2017). Pathobiology and diagnosis of multiple myeloma. Semin. Oncol. Nurs..

[3] Key Statistics About Multiple Myeloma. *American Cancer Society*. https://www.cancer.org/cancer/multiplemyeloma/detailedguide/multiple-myeloma-key-statistics. January 8, 2020; Accessed March 4 (2020).

[4] Multiple myeloma. https://www.cancer.net/cancer-types/multiple-myeloma

[5] Shah, D. & Seiter, K. *Multiple myeloma*. https://emedicine.medscape.com/article/204369-overview#a4.

[6] Palumbo A (2015). Revised international staging system for multiple myeloma: A report from international myeloma working group. J. Clin. Oncol..

[7] González-Calle V (2018). Evaluation of revised international staging system (R-ISS) for transplant-eligible multiple myeloma patients. Ann. Hematol..

[8] Greipp PR (2005). International staging system for multiple myeloma. J. Clin. Oncol..

[9] Bringhen S, De Wit E, Dimopoulos MA (2017). New agents in multiple myeloma: An examination of safety profiles. Clin. Lymphoma Myeloma Leuk..

[10] Anwer F (2019). Future of personalized therapy targeting aberrant signaling pathways in multiple myeloma. Clin. Lymphoma Myeloma Leuk..

[11] Siegel RL, Miller KD, Jemal A (2019). Cancer statistics, 2019. CA Cancer J. Clin..

[12] Beger RD (2013). A review of applications of metabolomics in cancer. Metabolites.

[13] Chanukuppa V (2018). Current understanding of the potential of proteomics and metabolomics approaches in cancer chemoresistance: A focus on multiple myeloma. Curr. Top. Med. Chem..

[14] Steiner N (2018). The metabolomic plasma profile of myeloma patients are considerably different from healthy subjects and reveals potential new therapeutic targets. PLoS ONE.

[15] Puchades-Carrasco L (2013). Multiple myeloma patients have a specific serum metabolomic profile that changes after achieving complete remission. Clin. Cancer Res..

[16] Hanahan D, Weinberg RA (2011). Hallmarks of cancer: The next generation. Cell.

[17] Phang JM (2019). Proline metabolism in cell regulation and cancer biology: Recent advances and hypotheses. Antioxid. Redox. Signal..

[18] Armitage EG, Ciborowski M (2017). applications of metabolomics in cancer studies. Adv. Exp. Med. Biol..

[19] Kaushik AK, DeBerardinis RJ (2018). Applications of metabolomics to study cancer metabolism. Biochim. Biophys. Acta Rev. Cancer..

[20] Sun L (2018). Metabolic reprogramming for cancer cells and their microenvironment: Beyond the Warburg Effect. Biochim. Biophys. Acta Rev. Cancer..

[21] Du H (2018). Analysis of the metabolic characteristics of serum samples in patients with multiple myeloma. Front. Pharmacol..

[22] Rizzieri D, Paul B, Kang Y (2019). Metabolic alterations, and the potential for targeting metabolic pathways in the treatment of multiple myeloma. J. Cancer Metastasis Treat..

[23] Ludwig C (2015). Alterations in bone marrow metabolism are an early and consistent feature during the development of MGUS and multiple myeloma. Blood Cancer J..

[24] Li S, Gao D, Jiang Y (2019). Function, detection and alteration of acylcarnitine metabolism in hepatocellular carcinoma. Metabolites.

[25] Schooneman MG, Vaz FM, Houten SM, Soeters MR (2013). Acylcarnitines: Reflecting or inflicting insulin resistance?. Diabetes.

[26] Xiang L (2017). Comprehensive analysis of acylcarnitine species in db/db mouse using a novel method of high-resolution parallel reaction monitoring reveals widespread metabolic dysfunction induced by diabetes. Anal. Chem..

[27] # 231680 Multiple Acyl-CoA Dehydrogenase Deficiency; MADD. https://www.omim.org/entry/231680.

[28] Grünert SC (2014). Clinical and genetical heterogeneity of late-onset multiple acyl-coenzyme A dehydrogenase deficiency. Orphanet J. Rare Dis..

[29] Sugai F (2012). Adult-onset multiple acyl CoA dehydrogenation deficiency associated with an abnormal isoenzyme pattern of serum lactate dehydrogenase. Neuromuscul. Disord..

[30] Bajpai R (2020). Electron transport chain activity is a predictor and target for venetoclax sensitivity in multiple myeloma. Nat. Commun.

[31] Frerman, F.E., Goodman, S.I. Defects of Electron Transfer Flavoprotein and Electron Transfer Flavoprotein-Ubiquinone Oxidoreductase: Glutaric Acidemia Type II. In *The Metabolic and Molecular Bases of Inherited Disease* (Scriver, C. R., Beaudet, A. L., Sly, W. S., Valle, D., Childs, B., Kinzler, K. W., and Vogelstein, B., eds.) (McGraw-Hill, New York, 2001).

[32] Wettersten HI (2015). Grade-dependent metabolic reprogramming in kidney cancer revealed by combined proteomics and metabolomics analysis. Cancer Res..

[33] Nakagawa H (2018). Lipid metabolic reprogramming in hepatocellular carcinoma. Cancers (Basel).

[34] Fujiwara N (2018). CPT2 downregulation adapts HCC to lipid-rich environment and promotes carcinogenesis via acylcarnitine accumulation in obesity. Gut.

[35] Wang YT (2018). Carnitine palmitoyltransferase 1C regulates cancer cell senescence through mitochondria-associated metabolic reprograming. Cell Death Differ..

[36] Harayama T, Riezman H (2018). Understanding the diversity of membrane lipid composition. Nat. Rev. Mol. Cell. Biol..

[37] Huang C, Freter C (2015). Lipid metabolism, apoptosis, and cancer therapy. Int. J. Mol. Sci..

[38] Satoh O (1990). Lipid composition of hepatitis B virus surface antigen particles and the particle-producing human hepatoma cell lines. J. Lipid Res..

[39] Mohamed A (2020). Concurrent lipidomics and proteomics on malignant plasma cells from multiple myeloma patients: Probing the lipid metabolome. PLoS ONE.

[40] Gibellini F, Smith TK (2010). The Kennedy pathway–De novo synthesis of phosphatidylethanolamine and phosphatidylcholine. IUBMB Life.

[41] Ogretmen B (2018). Sphingolipid metabolism in cancer signalling and therapy. Nat. Rev. Cancer.

[42] Seow HF (2004). Hartnup disorder is caused by mutations in the gene encoding the neutral amino acid transporter SLC6A19. Nat. Genet..

[43] Gullà A (2018). Protein arginine methyltransferase 5 has prognostic relevance and is a druggable target in multiple myeloma. Leukemia.

[44] Sarrouilhe D, Mesnil M (2019). Serotonin and human cancer: A critical view. Biochimie.

[45] Ocio EM (2006). Serotonin receptor antagonists have an in vitro and in vivo anti-myeloma effect that is mainly mediated by caspase dependent apoptosis. Blood.

[46] Chattopadhyay S (2019). Genome-wide interaction and pathway-based identification of key regulators in multiple myeloma. Commun. Biol..

[47] Zhang JZ, Henning SM, Swendseid ME (1993). Poly (ADP-ribose) polymerase activity and DNA strand breaks are affected in tissues of niacin-deficient rats. J. Nutr..

[48] Weidele K, Kunzmann A, Schmitz M, Beneke S, Bürkle A (2010). Ex vivo supplementation with nicotinic acid enhances cellular poly (ADP-ribosyl) ation and improves cell viability in human peripheral blood mononuclear cells. Biochem. Pharmacol..

[49] Stone TW (2001). Kynurenines in the CNS: From endogenous obscurity to therapeutic importance. Prog. Neurobiol..

[50] Müller-Thomas C, Heider M, Piontek G (2020). Prognostic value of indoleamine 2,3 dioxygenase in patients with higher-risk myelodysplastic syndromes treated with azacytidine. Br. J. Haematol..

[51] Wakabayashi Y, Yamada E, Hasegawa T, Yamada R (1991). Enzymological evidence for the indispensability of small intestine in the synthesis of arginine from glutamate I Pyrroline-5-carboxylate synthase. Arch. Biochem. Biophys..

[52] Gosselin KB (2014). Serum citrulline as a biomarker of gastrointestinal function during hematopoietic cell transplantation (CONTROLST) in children. J. Pediatr. Gastroenterol. Nutr..

[53] Jones JW (2015). Citrulline as a biomarker in the non-human primate total- and partial-body irradiation models: Correlation of circulating citrulline to acute and prolonged gastrointestinal injury. Health Phys..

[54] Rashidi A (2018). Pre-transplant serum citrulline predicts acute graft-versus-host disease. Biol. Blood Marrow Transplant..

[55] Rajkumar SV (2016). Updated diagnostic criteria and staging system for multiple myeloma. Am. Soc. Clin. Oncol. Educ. Book..

[56] Illig T (2010). A genome-wide perspective of genetic variation in human metabolism. Nat. Genet..

[57] Suhre K, Shin SY, Petersen AK (2011). Human metabolic individuality in biomedical and pharmaceutical research. Nature.

[58] Chong J (2019). MetaboAnalyst 4.0 for comprehensive and integrative metabolomics data analysis. Curr. Protoc. Bioinform..

[59] Xu L, Jia S, Li H (2018). Characterization of circulating tumor cells in newly diagnosed breast cancer. Oncol. Lett..

[60] Lin TY (2017). Incidence of abnormal liver biochemical tests in hyperthyroidism. Clin. Endocrinol. (Oxf).

[61] Raffler J (2015). Genome-Wide Association Study with Targeted and Non-targeted NMR Metabolomics Identifies 15 Novel Loci of Urinary Human Metabolic Individuality. PLoS Genet..

[62] Krumsiek J (2012). Mining the unknown: A systems approach to metabolite identification combining genetic and metabolic information. PLoS Genet..

[63] Gieger C, Geistlinger L, Altmaier E (2008). Genetics meets metabolomics: A genome-wide association study of metabolite profiles in human serum. PLoS Genet..

[64] Bartel J (2015). The human blood metabolome–transcriptome interface. PLoS Genet..

[65] Altmaier E (2008). Bioinformatics analysis of targeted metabolomics–uncovering old and new tales of diabetic mice under medication. Endocrinology.

[66] Janečková H (2012). Targeted metabolomic analysis of plasma samples for the diagnosis of inherited metabolic disorders. J. Chromatogr. A.

[67] da Silva I (2018). Inborn-like errors of metabolism are determinants of breast cancer risk, clinical response and survival: A study of human biochemical individuality. Oncotarget.

