# Supplementary material for: Biochemical phenotyping of multiple myeloma patients at diagnosis reveals a disorder of mitochondrial complexes I and II and a Hartnup-like disturbance as underlying conditions, also influencing different stages of the disease
Source: Sci Rep. 2020 Dec 14;10:21836. doi: 10.1038/s41598-020-75862-4 (PMC7736334; doi:10.1038/s41598-020-75862-4)
Supplement: Supplementary file 1 — Supplementary Table Legends. [file 41598_2020_75862_MOESM1_ESM.pptx]

## Slide 1
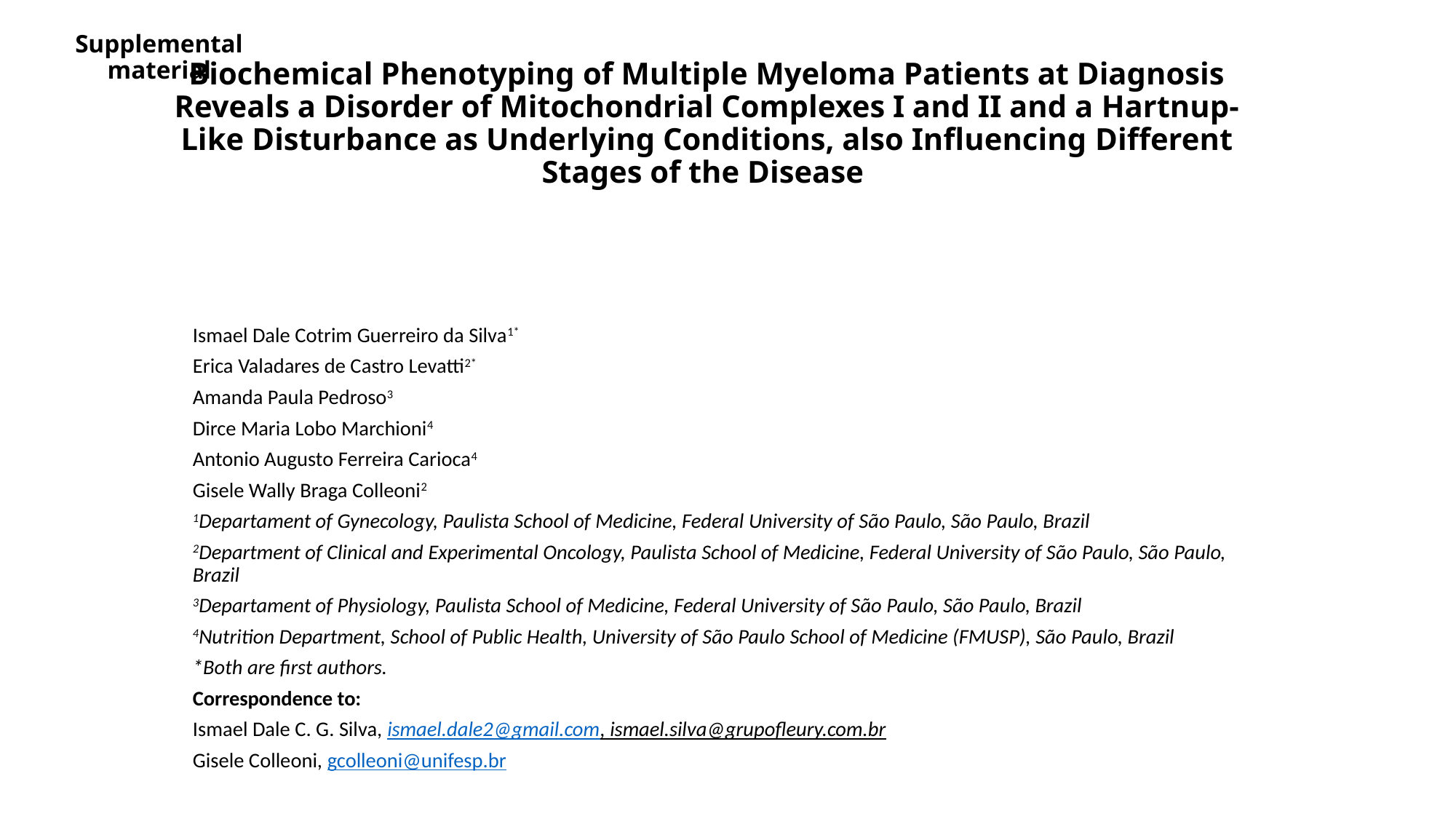

Supplemental material
# Biochemical Phenotyping of Multiple Myeloma Patients at Diagnosis Reveals a Disorder of Mitochondrial Complexes I and II and a Hartnup-Like Disturbance as Underlying Conditions, also Influencing Different Stages of the Disease
Ismael Dale Cotrim Guerreiro da Silva1*
Erica Valadares de Castro Levatti2*
Amanda Paula Pedroso3
Dirce Maria Lobo Marchioni4
Antonio Augusto Ferreira Carioca4
Gisele Wally Braga Colleoni2
1Departament of Gynecology, Paulista School of Medicine, Federal University of São Paulo, São Paulo, Brazil
2Department of Clinical and Experimental Oncology, Paulista School of Medicine, Federal University of São Paulo, São Paulo, Brazil
3Departament of Physiology, Paulista School of Medicine, Federal University of São Paulo, São Paulo, Brazil
4Nutrition Department, School of Public Health, University of São Paulo School of Medicine (FMUSP), São Paulo, Brazil
*Both are first authors.
Correspondence to:
Ismael Dale C. G. Silva, ismael.dale2@gmail.com, ismael.silva@grupofleury.com.br
Gisele Colleoni, gcolleoni@unifesp.br

## Slide 2
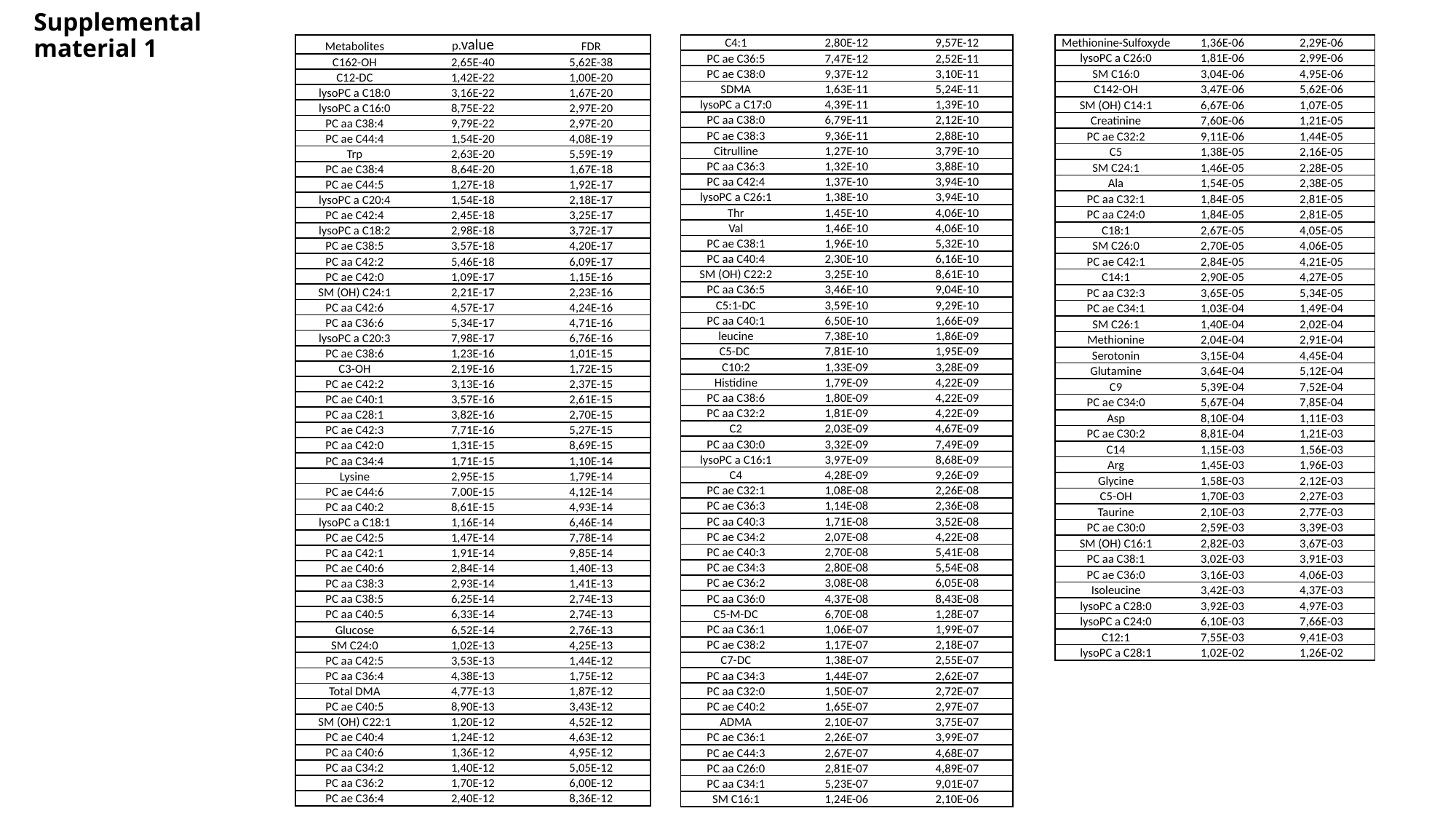

# Supplemental material 1
| Metabolites | p.value | FDR |
| --- | --- | --- |
| C162-OH | 2,65E-40 | 5,62E-38 |
| C12-DC | 1,42E-22 | 1,00E-20 |
| lysoPC a C18:0 | 3,16E-22 | 1,67E-20 |
| lysoPC a C16:0 | 8,75E-22 | 2,97E-20 |
| PC aa C38:4 | 9,79E-22 | 2,97E-20 |
| PC ae C44:4 | 1,54E-20 | 4,08E-19 |
| Trp | 2,63E-20 | 5,59E-19 |
| PC ae C38:4 | 8,64E-20 | 1,67E-18 |
| PC ae C44:5 | 1,27E-18 | 1,92E-17 |
| lysoPC a C20:4 | 1,54E-18 | 2,18E-17 |
| PC ae C42:4 | 2,45E-18 | 3,25E-17 |
| lysoPC a C18:2 | 2,98E-18 | 3,72E-17 |
| PC ae C38:5 | 3,57E-18 | 4,20E-17 |
| PC aa C42:2 | 5,46E-18 | 6,09E-17 |
| PC ae C42:0 | 1,09E-17 | 1,15E-16 |
| SM (OH) C24:1 | 2,21E-17 | 2,23E-16 |
| PC aa C42:6 | 4,57E-17 | 4,24E-16 |
| PC aa C36:6 | 5,34E-17 | 4,71E-16 |
| lysoPC a C20:3 | 7,98E-17 | 6,76E-16 |
| PC ae C38:6 | 1,23E-16 | 1,01E-15 |
| C3-OH | 2,19E-16 | 1,72E-15 |
| PC ae C42:2 | 3,13E-16 | 2,37E-15 |
| PC ae C40:1 | 3,57E-16 | 2,61E-15 |
| PC aa C28:1 | 3,82E-16 | 2,70E-15 |
| PC ae C42:3 | 7,71E-16 | 5,27E-15 |
| PC aa C42:0 | 1,31E-15 | 8,69E-15 |
| PC aa C34:4 | 1,71E-15 | 1,10E-14 |
| Lysine | 2,95E-15 | 1,79E-14 |
| PC ae C44:6 | 7,00E-15 | 4,12E-14 |
| PC aa C40:2 | 8,61E-15 | 4,93E-14 |
| lysoPC a C18:1 | 1,16E-14 | 6,46E-14 |
| PC ae C42:5 | 1,47E-14 | 7,78E-14 |
| PC aa C42:1 | 1,91E-14 | 9,85E-14 |
| PC ae C40:6 | 2,84E-14 | 1,40E-13 |
| PC aa C38:3 | 2,93E-14 | 1,41E-13 |
| PC aa C38:5 | 6,25E-14 | 2,74E-13 |
| PC aa C40:5 | 6,33E-14 | 2,74E-13 |
| Glucose | 6,52E-14 | 2,76E-13 |
| SM C24:0 | 1,02E-13 | 4,25E-13 |
| PC aa C42:5 | 3,53E-13 | 1,44E-12 |
| PC aa C36:4 | 4,38E-13 | 1,75E-12 |
| Total DMA | 4,77E-13 | 1,87E-12 |
| PC ae C40:5 | 8,90E-13 | 3,43E-12 |
| SM (OH) C22:1 | 1,20E-12 | 4,52E-12 |
| PC ae C40:4 | 1,24E-12 | 4,63E-12 |
| PC aa C40:6 | 1,36E-12 | 4,95E-12 |
| PC aa C34:2 | 1,40E-12 | 5,05E-12 |
| PC aa C36:2 | 1,70E-12 | 6,00E-12 |
| PC ae C36:4 | 2,40E-12 | 8,36E-12 |
| C4:1 | 2,80E-12 | 9,57E-12 |
| --- | --- | --- |
| PC ae C36:5 | 7,47E-12 | 2,52E-11 |
| PC ae C38:0 | 9,37E-12 | 3,10E-11 |
| SDMA | 1,63E-11 | 5,24E-11 |
| lysoPC a C17:0 | 4,39E-11 | 1,39E-10 |
| PC aa C38:0 | 6,79E-11 | 2,12E-10 |
| PC ae C38:3 | 9,36E-11 | 2,88E-10 |
| Citrulline | 1,27E-10 | 3,79E-10 |
| PC aa C36:3 | 1,32E-10 | 3,88E-10 |
| PC aa C42:4 | 1,37E-10 | 3,94E-10 |
| lysoPC a C26:1 | 1,38E-10 | 3,94E-10 |
| Thr | 1,45E-10 | 4,06E-10 |
| Val | 1,46E-10 | 4,06E-10 |
| PC ae C38:1 | 1,96E-10 | 5,32E-10 |
| PC aa C40:4 | 2,30E-10 | 6,16E-10 |
| SM (OH) C22:2 | 3,25E-10 | 8,61E-10 |
| PC aa C36:5 | 3,46E-10 | 9,04E-10 |
| C5:1-DC | 3,59E-10 | 9,29E-10 |
| PC aa C40:1 | 6,50E-10 | 1,66E-09 |
| leucine | 7,38E-10 | 1,86E-09 |
| C5-DC | 7,81E-10 | 1,95E-09 |
| C10:2 | 1,33E-09 | 3,28E-09 |
| Histidine | 1,79E-09 | 4,22E-09 |
| PC aa C38:6 | 1,80E-09 | 4,22E-09 |
| PC aa C32:2 | 1,81E-09 | 4,22E-09 |
| C2 | 2,03E-09 | 4,67E-09 |
| PC aa C30:0 | 3,32E-09 | 7,49E-09 |
| lysoPC a C16:1 | 3,97E-09 | 8,68E-09 |
| C4 | 4,28E-09 | 9,26E-09 |
| PC ae C32:1 | 1,08E-08 | 2,26E-08 |
| PC ae C36:3 | 1,14E-08 | 2,36E-08 |
| PC aa C40:3 | 1,71E-08 | 3,52E-08 |
| PC ae C34:2 | 2,07E-08 | 4,22E-08 |
| PC ae C40:3 | 2,70E-08 | 5,41E-08 |
| PC ae C34:3 | 2,80E-08 | 5,54E-08 |
| PC ae C36:2 | 3,08E-08 | 6,05E-08 |
| PC aa C36:0 | 4,37E-08 | 8,43E-08 |
| C5-M-DC | 6,70E-08 | 1,28E-07 |
| PC aa C36:1 | 1,06E-07 | 1,99E-07 |
| PC ae C38:2 | 1,17E-07 | 2,18E-07 |
| C7-DC | 1,38E-07 | 2,55E-07 |
| PC aa C34:3 | 1,44E-07 | 2,62E-07 |
| PC aa C32:0 | 1,50E-07 | 2,72E-07 |
| PC ae C40:2 | 1,65E-07 | 2,97E-07 |
| ADMA | 2,10E-07 | 3,75E-07 |
| PC ae C36:1 | 2,26E-07 | 3,99E-07 |
| PC ae C44:3 | 2,67E-07 | 4,68E-07 |
| PC aa C26:0 | 2,81E-07 | 4,89E-07 |
| PC aa C34:1 | 5,23E-07 | 9,01E-07 |
| SM C16:1 | 1,24E-06 | 2,10E-06 |
| Methionine-Sulfoxyde | 1,36E-06 | 2,29E-06 |
| --- | --- | --- |
| lysoPC a C26:0 | 1,81E-06 | 2,99E-06 |
| SM C16:0 | 3,04E-06 | 4,95E-06 |
| C142-OH | 3,47E-06 | 5,62E-06 |
| SM (OH) C14:1 | 6,67E-06 | 1,07E-05 |
| Creatinine | 7,60E-06 | 1,21E-05 |
| PC ae C32:2 | 9,11E-06 | 1,44E-05 |
| C5 | 1,38E-05 | 2,16E-05 |
| SM C24:1 | 1,46E-05 | 2,28E-05 |
| Ala | 1,54E-05 | 2,38E-05 |
| PC aa C32:1 | 1,84E-05 | 2,81E-05 |
| PC aa C24:0 | 1,84E-05 | 2,81E-05 |
| C18:1 | 2,67E-05 | 4,05E-05 |
| SM C26:0 | 2,70E-05 | 4,06E-05 |
| PC ae C42:1 | 2,84E-05 | 4,21E-05 |
| C14:1 | 2,90E-05 | 4,27E-05 |
| PC aa C32:3 | 3,65E-05 | 5,34E-05 |
| PC ae C34:1 | 1,03E-04 | 1,49E-04 |
| SM C26:1 | 1,40E-04 | 2,02E-04 |
| Methionine | 2,04E-04 | 2,91E-04 |
| Serotonin | 3,15E-04 | 4,45E-04 |
| Glutamine | 3,64E-04 | 5,12E-04 |
| C9 | 5,39E-04 | 7,52E-04 |
| PC ae C34:0 | 5,67E-04 | 7,85E-04 |
| Asp | 8,10E-04 | 1,11E-03 |
| PC ae C30:2 | 8,81E-04 | 1,21E-03 |
| C14 | 1,15E-03 | 1,56E-03 |
| Arg | 1,45E-03 | 1,96E-03 |
| Glycine | 1,58E-03 | 2,12E-03 |
| C5-OH | 1,70E-03 | 2,27E-03 |
| Taurine | 2,10E-03 | 2,77E-03 |
| PC ae C30:0 | 2,59E-03 | 3,39E-03 |
| SM (OH) C16:1 | 2,82E-03 | 3,67E-03 |
| PC aa C38:1 | 3,02E-03 | 3,91E-03 |
| PC ae C36:0 | 3,16E-03 | 4,06E-03 |
| Isoleucine | 3,42E-03 | 4,37E-03 |
| lysoPC a C28:0 | 3,92E-03 | 4,97E-03 |
| lysoPC a C24:0 | 6,10E-03 | 7,66E-03 |
| C12:1 | 7,55E-03 | 9,41E-03 |
| lysoPC a C28:1 | 1,02E-02 | 1,26E-02 |

## Slide 3
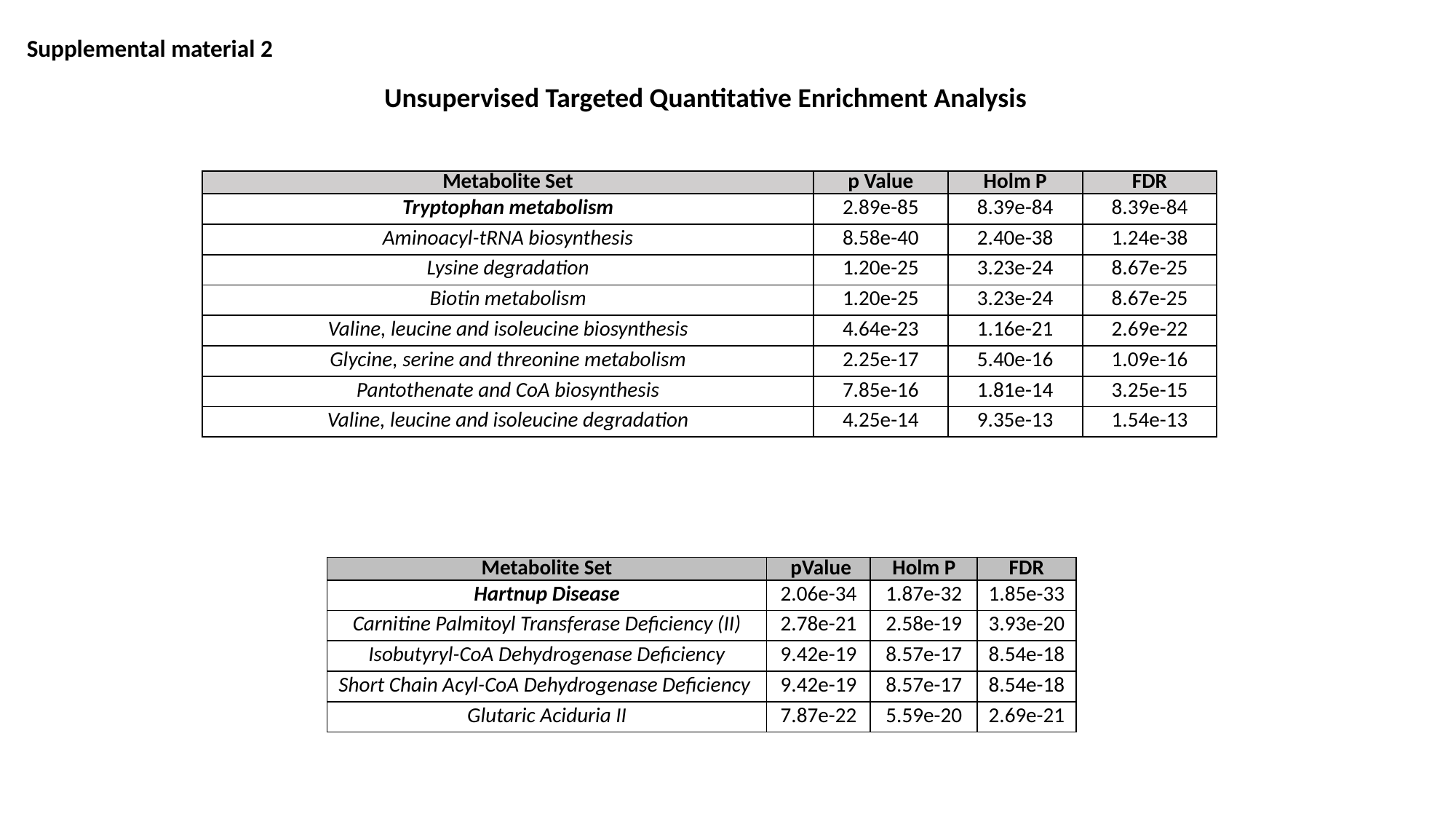

Supplemental material 2
Unsupervised Targeted Quantitative Enrichment Analysis
| Metabolite Set | p Value | Holm P | FDR |
| --- | --- | --- | --- |
| Tryptophan metabolism | 2.89e-85 | 8.39e-84 | 8.39e-84 |
| Aminoacyl-tRNA biosynthesis | 8.58e-40 | 2.40e-38 | 1.24e-38 |
| Lysine degradation | 1.20e-25 | 3.23e-24 | 8.67e-25 |
| Biotin metabolism | 1.20e-25 | 3.23e-24 | 8.67e-25 |
| Valine, leucine and isoleucine biosynthesis | 4.64e-23 | 1.16e-21 | 2.69e-22 |
| Glycine, serine and threonine metabolism | 2.25e-17 | 5.40e-16 | 1.09e-16 |
| Pantothenate and CoA biosynthesis | 7.85e-16 | 1.81e-14 | 3.25e-15 |
| Valine, leucine and isoleucine degradation | 4.25e-14 | 9.35e-13 | 1.54e-13 |
| Metabolite Set | pValue | Holm P | FDR |
| --- | --- | --- | --- |
| Hartnup Disease | 2.06e-34 | 1.87e-32 | 1.85e-33 |
| Carnitine Palmitoyl Transferase Deficiency (II) | 2.78e-21 | 2.58e-19 | 3.93e-20 |
| Isobutyryl-CoA Dehydrogenase Deficiency | 9.42e-19 | 8.57e-17 | 8.54e-18 |
| Short Chain Acyl-CoA Dehydrogenase Deficiency | 9.42e-19 | 8.57e-17 | 8.54e-18 |
| Glutaric Aciduria II | 7.87e-22 | 5.59e-20 | 2.69e-21 |

## Slide 4
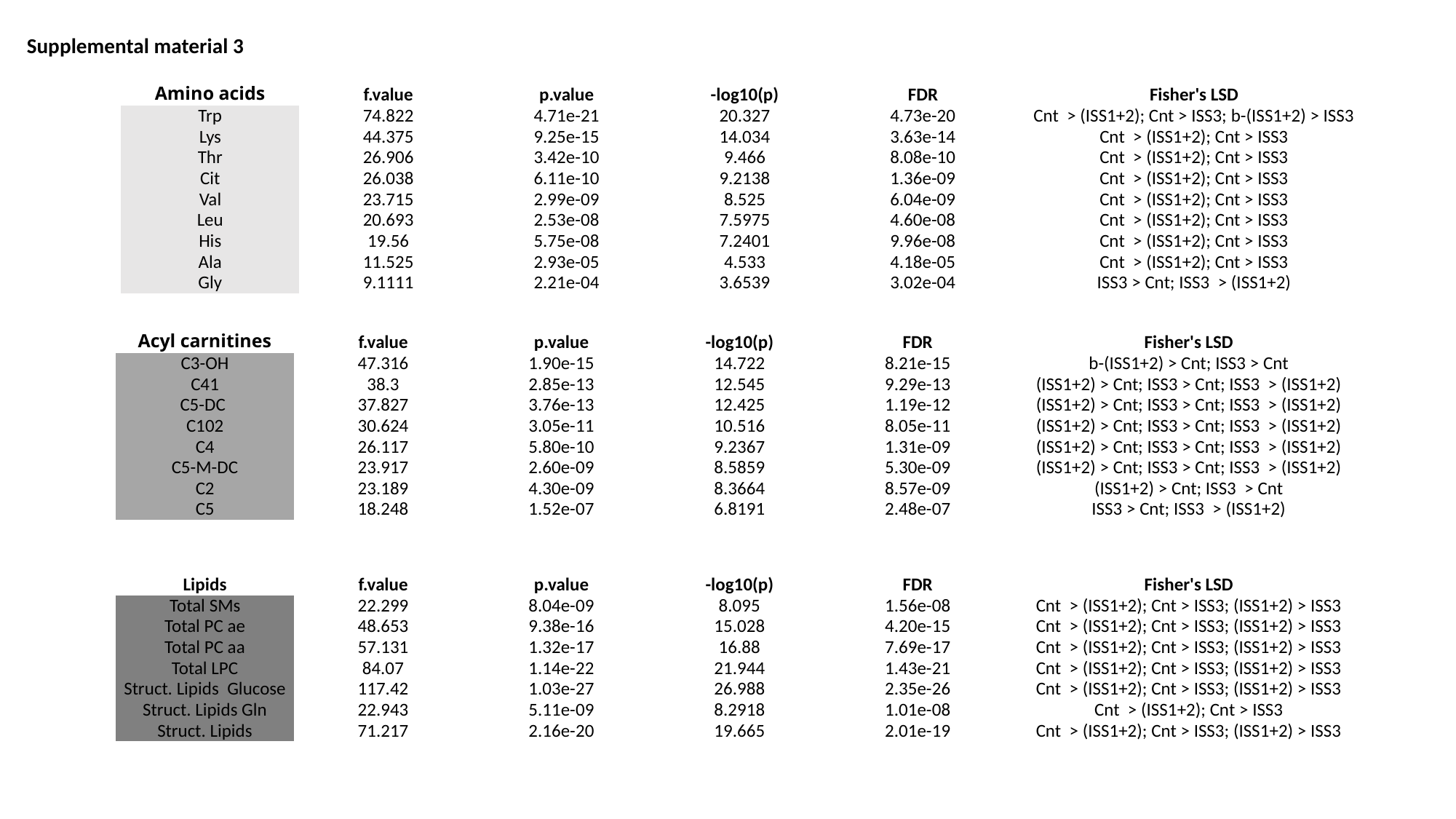

Supplemental material 3
| Amino acids | f.value | p.value | -log10(p) | FDR | Fisher's LSD |
| --- | --- | --- | --- | --- | --- |
| Trp | 74.822 | 4.71e-21 | 20.327 | 4.73e-20 | Cnt > (ISS1+2); Cnt > ISS3; b-(ISS1+2) > ISS3 |
| Lys | 44.375 | 9.25e-15 | 14.034 | 3.63e-14 | Cnt > (ISS1+2); Cnt > ISS3 |
| Thr | 26.906 | 3.42e-10 | 9.466 | 8.08e-10 | Cnt > (ISS1+2); Cnt > ISS3 |
| Cit | 26.038 | 6.11e-10 | 9.2138 | 1.36e-09 | Cnt > (ISS1+2); Cnt > ISS3 |
| Val | 23.715 | 2.99e-09 | 8.525 | 6.04e-09 | Cnt > (ISS1+2); Cnt > ISS3 |
| Leu | 20.693 | 2.53e-08 | 7.5975 | 4.60e-08 | Cnt > (ISS1+2); Cnt > ISS3 |
| His | 19.56 | 5.75e-08 | 7.2401 | 9.96e-08 | Cnt > (ISS1+2); Cnt > ISS3 |
| Ala | 11.525 | 2.93e-05 | 4.533 | 4.18e-05 | Cnt > (ISS1+2); Cnt > ISS3 |
| Gly | 9.1111 | 2.21e-04 | 3.6539 | 3.02e-04 | ISS3 > Cnt; ISS3 > (ISS1+2) |
| Acyl carnitines | f.value | p.value | -log10(p) | FDR | Fisher's LSD |
| --- | --- | --- | --- | --- | --- |
| C3-OH | 47.316 | 1.90e-15 | 14.722 | 8.21e-15 | b-(ISS1+2) > Cnt; ISS3 > Cnt |
| C41 | 38.3 | 2.85e-13 | 12.545 | 9.29e-13 | (ISS1+2) > Cnt; ISS3 > Cnt; ISS3 > (ISS1+2) |
| C5-DC | 37.827 | 3.76e-13 | 12.425 | 1.19e-12 | (ISS1+2) > Cnt; ISS3 > Cnt; ISS3 > (ISS1+2) |
| C102 | 30.624 | 3.05e-11 | 10.516 | 8.05e-11 | (ISS1+2) > Cnt; ISS3 > Cnt; ISS3 > (ISS1+2) |
| C4 | 26.117 | 5.80e-10 | 9.2367 | 1.31e-09 | (ISS1+2) > Cnt; ISS3 > Cnt; ISS3 > (ISS1+2) |
| C5-M-DC | 23.917 | 2.60e-09 | 8.5859 | 5.30e-09 | (ISS1+2) > Cnt; ISS3 > Cnt; ISS3 > (ISS1+2) |
| C2 | 23.189 | 4.30e-09 | 8.3664 | 8.57e-09 | (ISS1+2) > Cnt; ISS3 > Cnt |
| C5 | 18.248 | 1.52e-07 | 6.8191 | 2.48e-07 | ISS3 > Cnt; ISS3 > (ISS1+2) |
| Lipids | f.value | p.value | -log10(p) | FDR | Fisher's LSD |
| --- | --- | --- | --- | --- | --- |
| Total SMs | 22.299 | 8.04e-09 | 8.095 | 1.56e-08 | Cnt > (ISS1+2); Cnt > ISS3; (ISS1+2) > ISS3 |
| Total PC ae | 48.653 | 9.38e-16 | 15.028 | 4.20e-15 | Cnt > (ISS1+2); Cnt > ISS3; (ISS1+2) > ISS3 |
| Total PC aa | 57.131 | 1.32e-17 | 16.88 | 7.69e-17 | Cnt > (ISS1+2); Cnt > ISS3; (ISS1+2) > ISS3 |
| Total LPC | 84.07 | 1.14e-22 | 21.944 | 1.43e-21 | Cnt > (ISS1+2); Cnt > ISS3; (ISS1+2) > ISS3 |
| Struct. Lipids Glucose | 117.42 | 1.03e-27 | 26.988 | 2.35e-26 | Cnt > (ISS1+2); Cnt > ISS3; (ISS1+2) > ISS3 |
| Struct. Lipids Gln | 22.943 | 5.11e-09 | 8.2918 | 1.01e-08 | Cnt > (ISS1+2); Cnt > ISS3 |
| Struct. Lipids | 71.217 | 2.16e-20 | 19.665 | 2.01e-19 | Cnt > (ISS1+2); Cnt > ISS3; (ISS1+2) > ISS3 |
