# Supplementary material for: Biochemical phenotyping of multiple myeloma patients at diagnosis reveals a disorder of mitochondrial complexes I and II and a Hartnup-like disturbance as underlying conditions, also influencing different stages of the disease
Source: Sci Rep. 2020 Dec 14;10:21836. doi: 10.1038/s41598-020-75862-4 (PMC7736334; doi:10.1038/s41598-020-75862-4)
Supplement: Supplementary file 2 — Supplementary Tables. [file 41598_2020_75862_MOESM2_ESM.docx]

**Supplemental Material 1.** P values (t test) and false discovery ratio (FDR) of metabolites presenting differences between multiple myeloma and control groups.

**Supplemental Material 2.** Unsupervised Targeted Quantitative Enrichment Analysis (p value, Holm P, FDR).

**Supplemental Material 3.** Main analyzed metabolites p value, FDR, and Fisher LSD.
